# Next-Generation Anticancer Peptides: Engineering, Nanotheranostics and Clinical Translation

**DOI:** 10.7150/ntno.133576

**Published:** 2026-04-16

**Authors:** Abhishesh Kumar Mehata, Shinsuke Fukui, Yoshihiro Izumiya

**Affiliations:** 1 Department of Dermatology, School of Medicine, University of California, Davis (UC Davis), 3301 C-street, Sacramento, CA 95816, USA.; 2 Department of Biochemistry and Molecular Medicine, School of Medicine, UC Davis, 4645 2 nd Avenue, Sacramento, CA 95817, USA.; 3 UC Davis Comprehensive Cancer Center, 2279 45 th St, Sacramento, CA 95817, USA.

**Keywords:** anticancer peptide, targeted cancer therapy, drug delivery, theranostics, clinical translation

## Abstract

Anticancer peptides (ACPs) have emerged as a transformative class of next-generation therapeutics that bridge molecular precision with multifunctional tunability. Unlike many conventional small molecule chemotherapeutics, ACPs offer intrinsic selectivity toward malignant cells through preferential membrane targeting, immunomodulation, and disruption of oncogenic signalling pathways. Advances in peptide engineering, including sequence optimization, incorporation of non-natural amino acids, cyclization, stapling, PEGylation, and structure-activity relationship-guided refinement, have substantially improved their stability, potency, and pharmacokinetic performance. Parallel progress in nanotechnology has further expanded the translational potential of ACPs by enabling controlled release, cancer cell specific targeting, and multimodal theranostic integration. Lipid nanoparticles, solid lipid nanoparticles, polymeric systems, dendrimers, mesoporous silica nanoparticles, and stimuli-responsive platforms now provide multiple and combinatorial strategies to overcome biological barriers, enhance intracellular delivery, and minimize systemic toxicity. Emerging concepts such as enzyme-activated nanocarriers, ligand-directed precision delivery, and light- or pH-responsive systems are redefining and energizing the spatial and temporal control of peptide therapeutics research fields. Despite encouraging preclinical and early clinical progress, including FDA-approved peptide-based agents and peptide receptor radionuclide therapies, challenges related to stability, immunogenicity, manufacturing scalability, and regulatory harmonization remain significant. This review highlights current advances in ACP discovery, molecular engineering, and nanotheranostic integration, and outlines a roadmap for advancing peptide-based precision oncology. Collectively, next-generation ACP platforms hold promise to reshape cancer therapy by integrating targeted cytotoxicity, immune activation, and real-time imaging within a single modular framework.

## 1. Introduction

Cancer remains a leading global health challenge in 2026, with over 2.1 million new cases projected to be diagnosed in the USA, and over ~626,000 deaths expected, unfortunately 1, 2. Conventional chemotherapy has limitations in treating cancer, as the tumor can often develop resistance to chemotherapy, leading to reduced therapeutic efficacy and increasing adverse effects after therapy 3. Many small peptides have been discovered to have anti-cancer activity in a variety of species, along with the development of molecular science. These peptides also have the ability to control the immune response and eradicate pathogenic organisms and cancer cells. Anticancer peptides (ACPs) are cationic low-molecular-weight peptides with anti-tumor action that have emerged as a result of the collection of both structural and functional data 4. Before it was initially identified as a powerful anti-cancer drug in 1985, cationic peptides derived from a variety of species were actually evaluated for their antibacterial properties and investigated as such. ACPs are inexpensive to produce and simple to alter with advanced solid-phase synthesis technologies 5. ACPs also offer advantages that include excellent affinity, great accessibility, and ease of customisation. An anticancer drug should ideally target only cancer cells, but not healthy normal cells 6. The distribution and abundance of cell membrane proteins often distinguish normal cells from malignant cells. Accordingly, many anticancer peptides specifically bind to and cause tumor cells to undergo apoptosis and/or necrosis by membrane rupture or pore formation. Anticancer drugs are biologically tailored substances that engage with certain biological targets on cancer cells, either with or without "targeting moiety"^7^. In addition to molecules that specifically target certain proteins, drug delivery to the surface of cancer cells via an affinity peptide also offered a high degree of selectivity and the capacity to attach to different targeted medications 8. These peptide characteristics can be utilised in both molecularly targeted therapies and targeting ligands to specifically target cancerous cells 9. ACPs offer high specificity, selectivity and potency but suffer from major pharmacokinetic limitations that include rapid enzymatic degradation, short plasma half-life and poor membrane permeability. Further, clinical barriers that limit ACPs' activity include low oral bioavailability, which requires parenteral injection and limited intracellular targeting 10.

The literature included in this review has been collected from major databases, including PubMed, Scopus and Web of Science. The time frame for the literature is approximately 2010 to 2025, with a primary emphasis on the studies published within last 5-10 years, presenting recent advances in ACPs research. This review highlights recent progress in ACPs discovery, rational molecular engineering, nanotheranostic integration, and clinical development, while proposing strategic directions to accelerate their translation into precision oncology. Overall, the next-generation ACPs system represents an exciting platform in cancer management by unifying selective tumor cytotoxicity, immunomodulatory activation, and image-guided therapeutic monitoring within a versatile and modular therapeutic architecture.

## 2. Engineering and Peptide Development

Owing to improved methods for manufacture, alteration, and analysis, peptide drug discovery has advanced significantly during the past ten years. Biochemical, molecular biological, and microbiological approaches are used to identify peptides. Innovative design and advanced delivery techniques have also helped researchers to overcome the inherent limitations of peptides and progress in the field of cancer therapy. Numerous peptides, both natural and engineered, have been discovered and investigated in several therapeutic domains 11.

### 2.1. Sources of Anticancer Peptides

#### 2.1.1. Natural (animal, plant, viral, microbial, marine)

Novel therapeutic peptide medications are mostly derived from natural sources. Over half of the globe's biodiversity is found in the sea, which makes up around 71% of the planet's surface. Many bioactive peptides with anticancer activity have been found in marine creatures, namely in fish, sponges, molluscs, marine arthropods, and ascidians. It has also been demonstrated that several known amino acids from terrestrial species, such as arthropods that live on land, amphibians, reptiles, and mammals, have a wide range of potential uses as innovative anti-tumor medications or supplementary therapeutic approaches for the treatment of malignancy 12.

In addition to the natural resource, recent studies recognized that viral and host protein interaction is an avenue to isolate a potent peptide sequence, which controls host protein functions 13-15. The idea comes from the fact that viruses depend on the host cell enzymes for their replication, and million years of co-evolution have shaped the viral protein to interact with specific proteins with higher affinity in order to effectively utilise the targeted cell host proteins in the presence of other cellular protein competitors. Other powerful approaches to identify high affinity peptide drugs, such as phage display 16, 17, one-beads-one peptide screening 18-21, and AI-assisted docking simulation 22-26, are predominantly reviewed by others 27-30. Following, we like to highlight a few examples of potent peptide sequences identified from basic virology in this section.

In an earlier study, Lee *et al*. identified FLICE-like inhibitory protein (FLIP) as a regulator of autophagy, which blocks LC3-Atg3 interaction and suppressed autophagosomes formation. Importantly, short peptides delivered from the DED1 α2 and DED2 α4 helical region were identified as conserved with the viral homolog of FLIP, and the identified small peptide has disrupted the FLIP-Atg3 binding, autophagy mediated cancer cells death in Kaposi's sarcoma-associated herpesvirus (KSHV)-associated lymphoma xenograft models 13.

**Figure 1:**
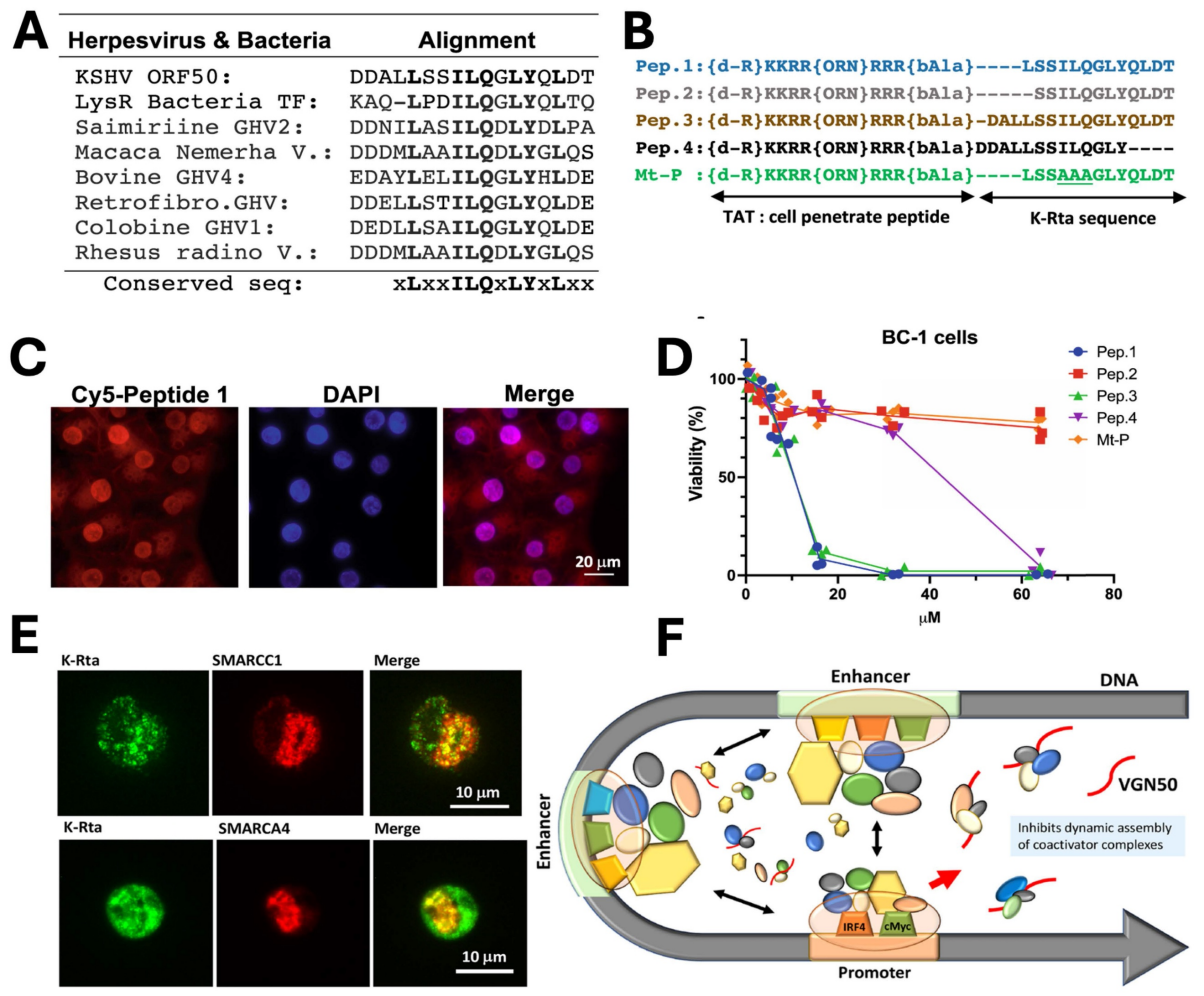
Discovery and mechanism of the VGN50, viral K-Rta derive peptide; A) Sequence alignment identifying the conserved K-Rta transactivation motif used for peptide design. B) Schematic of VGN50 incorporating the conserved sequence and TAT cell-penetrating domain. C) Fluorescence imaging confirming rapid nuclear uptake of VGN50. D) Dose-dependent reduction of PEL cell viability by VGN50 versus mutant control. E) Colocalization of K-Rta with SWI/SNF components in reactivating cells. F) Proposed model showing VGN50 trapping coactivator complexes and inhibiting MYC transcription. *Reproduced with permission from 14, Fig. 2, & Fig. 5 (©2021 Nature)*.

By adapting a similar concept, a novel cellular myelocytomatosis oncogene (MYC) targeting strategy was also reported by Shimoda *et al*., who developed VGN50, a small peptide derived from the intrinsically disordered transactivation domain of the KSHV Replication and trans-activation (K-Rta) protein. VGN50 was identified following proteomic analysis, which revealed that K-Rta recruits a large coactivator complex containing SWI/SNF components, NCOA2, and p300; sequence alignment of conserved regions within the K-Rta transactivation domain led to the design of cell-penetrating peptides **(Fig. 1A, B)**. Among these, Pep1 (renamed VGN50) showed potent cytotoxic activity in primary effusion lymphoma (PEL) cells, significantly reducing cell viability in a dose-dependent manner compared to mutant controls (**Fig. 1C, D**). Mechanistically, VGN50 acts as a decoy peptide that directly binds SWI/SNF subunits, sequestering the coactivator complex and preventing its recruitment to the MYC promoter and enhancer regions, resulting in decreased RNAPII occupancy, reduced H3K27ac enrichment, and suppression of MYC and MYC-target gene transcription. Immunofluorescence studies further demonstrated colocalization of K-Rta with SWI/SNF components during viral reactivation (**Fig. 1E**), supporting the biological and virological relevance of this interaction. A mechanistic model (**Fig. 1F**) proposes that VGN50 traps coactivator complexes away from cellular enhancers, thereby attenuating MYC-driven transcriptional programs. Importantly, VGN50 inhibited leukaemia and lymphoma cell growth both *in vitro* and in a PEL xenograft model, highlighting its potential as a peptide-based therapeutic strategy for MYC-dependent malignancies.

**Figure 2:**
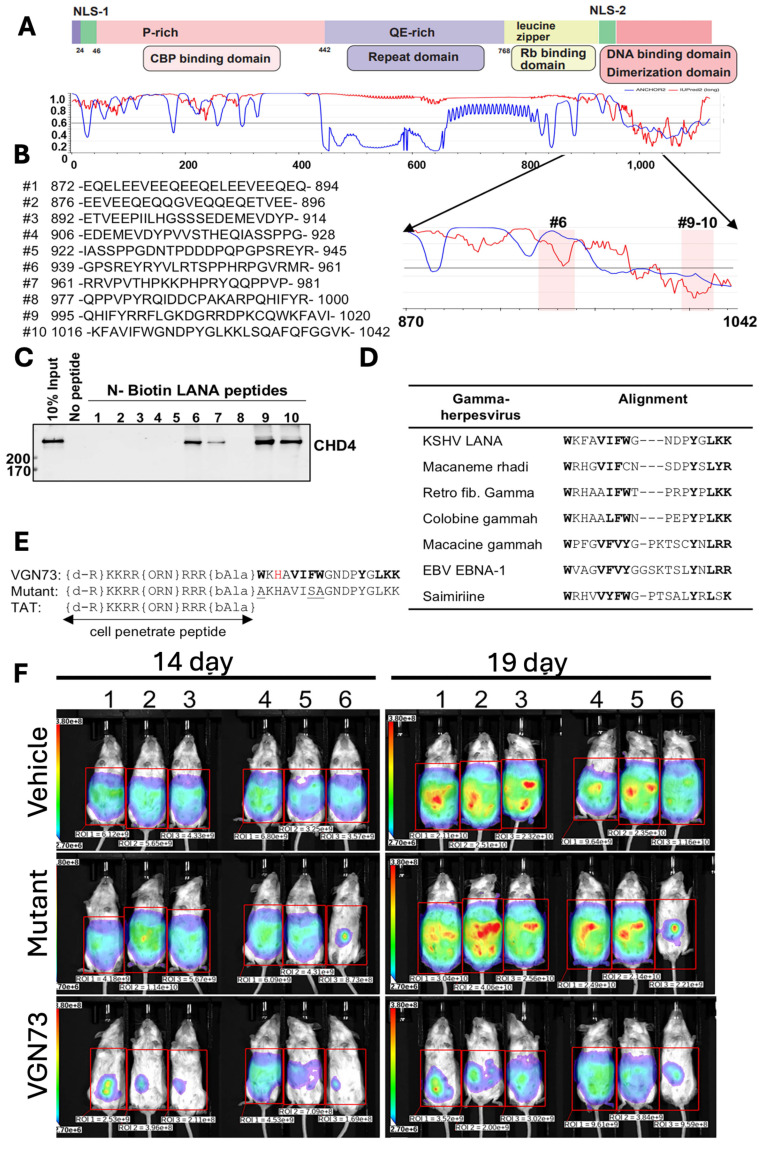
Development of VGN73 peptide and its antitumor efficacy in mouse model, **A-E)** Mapping of the CHD4-interacting region within KSHV LANA (aa 870-1042) using overlapping biotinylated peptides identified fragments (#9-10) that bind CHD4. Sequence alignment across γ-herpesviruses revealed conserved residues, guiding the rational design of the cell-penetrating peptide VGN73, with stabilizing modifications and a mutant control. F) In a BCBL-1 xenograft model, bioluminescence imaging demonstrated that VGN73 (10 mg/kg) markedly reduced tumor burden compared with vehicle and mutant peptide controls, confirming its *in vivo* antitumor activity. *Reproduced with permission from 15, Fig. 1, & Fig. 7 (© 2024 Elsevier).*

In another study, Miura *et al*., discovered the VGN73 peptide from the CHD4-interacting region of the KSHV latency-associated nuclear antigen (LANA). The VGN73 was discovered by first mapping the LANA-CHD4 interacting domain (aa 870-1042) and screening the overlapping biotinylated peptides, and then identifying the sequences (#9-10) that bound to CHD4 (**Fig. 2A-C**). Additionally, the conserved aromatic and basic residue was observed from sequence alignment across γ-herpesvirus homologs (**Fig. 2D**), which guided rational optimization into a shortened, cell-penetrating peptide containing a TAT motif and stability-enhancing modifications (**Fig. 2E**). VGN73 works by binding with the PHD domain of CHD4 with high affinity (~14 nM KD), promoting caspase-dependent CHD4 cleavage, inducing apoptosis and autophagy in the lymphoma cells. An *in vivo* study demonstrated that VGN73 has significantly inhibited primary effusion lymphoma (PEL) progression in a xenograft mouse model, which is depicted by reduced bioluminescence signal over time **(Fig. 2F).** Overall, VGN73, a virus-inspired natural peptide serves as a promising candidate for *in vivo* PEL therapy 15. We expect that more and more potent peptide sequences will be identified from viral-host protein interactions.

#### 2.1.2. Synthetic and Engineered Peptides

The synthetic peptides can be designed to mimic the activity of the naturally occurring peptides, and further modification leads to enhancing their anticancer activity and also stability 9. Synthetic ACPs possess several advantages over natural peptides, such as target specificity, enhancement of the activity and stability, reduced toxicity and immunogenicity, enhanced tumor penetration, etc. ACPs act by various mechanism that includes cancer cell membrane disruption, necrosis/apoptosis, preventing angiogenesis and metastasis, immunomodulation, and disruption in the cancer signalling pathway 31.

### 2.2. Mechanisms of Anticancer Action

#### 2.2.1. Membrane Disruption

The plasma membrane functions as a highly dynamic, selectively permeable barrier which is essential for maintaining cellular integrity and homeostasis. Numerous studies have shown that endogenous antimicrobial peptides destroy cancerous cells by rupturing the cell's membrane, even though the lipid bilayer is crucial for cell survival and physiological activities. These peptides selectively target negatively charged membrane constituents such as heparan sulphate, sialic acid, and phosphatidylserine (PS). A key difference between malignant and normal cells lies in the cell membrane lipid asymmetry. In normal cells, phosphatidylserine is preferentially confined to the inner leaflet of the plasma membrane, whereas the outer leaflet is composed of zwitterionic phospholipids such as phosphatidylcholine and sphingomyelin, resulting in relatively neutral charge. In contrast, cancer cells exhibit externalisation of the phosphatidylserine to the outer leaflet of the membrane, generating an overall negative charge to the cancer cells that selectively interacts with the positively charged anticancer peptides. This preferential binding ultimately leads to membrane destabilisation, pore formation, increased permeability, and subsequent cell lysis or apoptosis. Membrane disrupting peptide such as magainin II and melittin exert a rapid cytotoxic effect by direct destabilisation of the plasma membrane 32, 33.

#### 2.2.2. Induction of Apoptosis

Several ACPs induce cancer cell death via cell apoptosis. They mainly work by disrupting the mitochondrial membrane that leads to the release of cytochrome c and proapoptotic factors that stimulate the apoptotic pathway. The peptides KLAKLAK and Bax-derived peptides primarily induce apoptosis via mitochondrial dysfunction and activation of the intrinsic apoptotic signalling pathway 34, 35.

#### 2.2.3. Immunomodulation

ACPs also exert their anticancer activity by immunomodulatory mechanisms, either by stimulating or regulating components of the immune system to enhance the anticancer effect. These peptides can function as immune activators, immune adjuvants or tumor-induced immunosuppression^6^, ^31^. The length of immunomodulatory peptides varies; smaller peptides can produce transient immune responses, whereas longer peptide eliciting sustained and long-lasting antitumor immunity. The immunomodulatory activities of the ACPs can trigger the discharge of the danger signal from the dying cancer cells and activate chemokine genes, enhance T-cells immune response and inhibit T regulatory cells. Short peptides (8-12 amino acids) often bind to antigen-presenting cells and trigger CD8+ T cells. Long peptides (≥ 20 amino acids) can be presented by MHC I and II molecules, activating both CD8+ and CD4+ T cells. This dual activation contributes to a more robust and durable antitumor immune response. Unlike membranolytic peptides that directly damage plasma membrane integrity, immunomodulator peptides work by strengthening the host immune response. These peptides thus function as stimulators of adaptive immunity, which includes tumor-associated antigen vaccines or immune adjuvants. For example, anticancer peptides epitopes obtained from Wilms tumor 1^36^, Survivin 37 and NY-ESO-1^38^ antigen were predominantly studied as cancer vaccine antigens. These peptides function as immunogenic epitopes and are designed to stimulate tumor-specific CD8+ cytotoxic T lymphocytes response.

#### 2.2.4. Anti-angiogenic and Anti-metastatic Effects

Angiogenesis is required for tumor to grow and is associated with tumor metastasis. Growing tumors are in high demand for nutrients and oxygen supplies for their growth. Selective anti-cancer peptides inhibit the formation of new blood vessels and stop the spread of the tumor to distant organs 39. Other peptides also contribute to antiangiogenic and antimetastatic include angiostatin 40, tumstatin 41, and cilengitide 42. We have compiled the list of anticancer peptides, including their source, mechanism of action, and therapeutic applications in various types of cancer, as presented in **Table 1**. We also listed peptide drugs that are currently in clinical trials in **Table 2**.

### 2.3. Peptide Optimization Strategies

#### 2.3.1. Sequence Modification

Sequence modification represents a powerful technique to overcome the structural and functional limitations associated with the canonical set of 20 natural amino acids. Inclusion of non-natural amino acids in the development of anti-cancer peptides provides access to enhance stability, improve bioavailability and tailor physicochemical characteristics and hence expand the therapeutic capability of peptide-based anti-cancer drugs. A desirable method for immediate possession of customised peptides is being able to chemically modify individual amino acids within the framework of a complex peptide. This eliminates the requirement to make an orthogonally protected, unnatural amino acid modification, which usually calls for specialised knowledge of organic synthesis and time-consuming, multi-step procedures. Only a few amino acids have traditionally been used as handles for peptide functionalisation. However, the approaches addressed here leverage distinct, frequently underutilised amino acid functions within the context of increasingly complex peptide and protein scaffolds by strategically using a transition-metal mediator. Three primary types of approaches are addressed: C-H functionalization, decarboxylative couplings at α-COOH, Asp, and Glu, and heteroatom-mediated coupling at sulphur, selenium, and nitrogen. Proteins and peptides can be chemically modified in a variety of ways thanks to transition elements. Utilising biological groups for bioconjugation as well as structural modification has created new opportunities for retrosynthetic planning. For example, a single N-H bond in a protein can be precisely arylated, or carboxylic acids and even inactivated C- H bonds can function as unconventional handles for C-C bonds to form 75. The example includes R-lycosin-I 76 has arginine modification, which improved the anticancer activity, NRC-03 77 promoted apoptosis and oxidative stress in cancer cells compared to its native peptide.

#### 2.3.2. Cyclization, Stapling, and PEGylation

Peptides based therapeutics often need to be modified further in order to more closely resemble the biological peptide or protein fragment on which they were patterned or to include components that improve pharmacological performance. By using suitably derivatised amino acids, the majority of changes may be added either post-synthetically or during the peptide synthesis. Cyclisation, stapling, phosphorylation, or biotinylating, and PEGylation are a few frequent modifications in peptide production. Numerous naturally occurring peptides that exhibit intriguing biological action are cyclic peptides. In synthetic peptides, cyclisation is also employed to force a desirable shape, particularly when the peptide is derived from a segment of a much bigger peptide or protein. Sidechain-to-sidechain, terminus-to-sidechain, and terminus-to-terminus are the three ways that cyclic peptides can form. In every instance, cyclisation is usually carried out following the synthesis of the linear peptide 78, 79. Disulphide bridging of cysteine residues is the most prevalent kind of sidechain-to-sidechain cyclisation. By exposing two cysteine groups and oxidising them to generate the disulfide link, this cyclisation is initiated. Using selectively detachable sulfhydryl protective moieties allows for the selective formation of several cycles.

Cyclisation can be carried either on-resin prior to cleavage or in solution after cleavage from the resin. Two allylglycine residues cyclized via ring closure synthesis have been used to create analogues of disulfide bridging 80, 81. The functional configuration of the relevant region of the original protein is frequently lost when an engineered peptide is derived from a portion of a bigger peptide or protein. The peptide can take on an α-helical form by adding hydrocarbon bridges, sometimes known as clamps, among residues. When contrasted to their non-stapled counterparts, looped peptides can exhibit noticeably higher activity. Other looped peptides could be more effective in entering cells and withstanding hydrolysis by enzymes. Peptides' medicinal properties can be enhanced by adding polyethylene glycol (PEG) chains. The large PEG prevents proteolytic enzymes from breaking down the amino acid. Renal clearance is significantly reduced by a PEGylation of the peptide due to the hydrodynamic radius being larger than the typical cross section of glomerular capillaries. The peptide's functional half-life in the body is increased by these factors taken together. PEGylation may also have negative consequences. Although PEGylation prevents ACPs from enzymatic breaking, the PEG bulk may decrease the peptide's ability to connect to the intended receptor. The longer pharmacokinetic half-life of the PEGylated peptide often compensates for its decreased affinity. Therefore, optimization of PEG polymer size and attachment site is critical to achieving a balance between improved pharmacokinetics and maintained biological activity 82, 83.

#### 2.3.3. Structure-Activity Relationship (SAR) Studies

Structure-activity relationship (SAR) studies play a critical role in the rational design and optimisation of anticancer peptides. A large percentage of ACPs had 21-30 amino acids and were mostly constituted of glycine, lysine, and leucine, according to research and predictive analysis of the relationship between ACPs and SAR 84. Furthermore, a peptide's antitumor action is influenced by its amino acid residue based on the cationic, hydrophobic, and amphiphilic characteristics linked to helical structural formation. The inhibitory concentration (IC_50_) linked to rupture of the cancerous cell membrane is the main indicator of antitumor efficacy. Higher hydrophobicity peptides enter the hydrophobic centre of the cancerous cell's membranes and cause necrosis, which disrupts the cancer cell 85. ACPs demonstrate different structural features that govern their anti-cancer properties. In two-dimensional (2D) representation, ACPs are defined by their amino acid sequence, highlighting the distribution of cationic and hydrophobic residues. In three-dimensional (3D) structures, these peptides commonly adopt α-helical or β-sheet conformations, resulting in an amphipathic architecture with spatial separation of hydrophobic and hydrophilic domains. Such a structural arrangement supports selective interaction with the negatively charged cancer cell membrane that facilitates membrane disruption and permeability 86, 87. On both the polar and non-polar sides of α-helical peptides, a number of research efforts replace low hydrophobic and neutral or acidic amino acid residues with positively charged amino acid residues like lysine and leucine. Therefore, the toxic effects of tumor cells can be increased by high positively charged peptides with moderate hydrophobic properties. Free-form peptides do not fold in water; instead, they form an α-helix or β-sheet on the cell outer membrane by electrostatic contact 9. The secondary architecture of the peptides, such as peptide structural arrangement, is crucial for cell surface contact in addition to their physical characteristics. Peptide alignment can improve the activity of surfaces for specific contact with the membrane of cancerous cells. Membrane penetrating results from the interaction's angle, which causes unstable lipid packed on the malignant cell membrane.

## 3. Formulation and Drug Delivery Strategies

Although structural alteration in the peptide can enhance its stability and pharmacological properties, the clinical translation remains limited by its inherent physicochemical challenges. However, the majority of peptide medications are administered by injection because peptides are easily hydrolysed by gastrointestinal enzymes in the gut. To circumvent the problem, recent research has explored peptide drug delivery methods 10, 88. More promisingly, the combination of semaglutide and sodium N-[8-(2-hydroxybenzoyl amino)caprylate (SNAC) co-delivery system was authorised for oral treatment of type 2 diabetes. By reducing the effect of digestive enzymes, their co-delivery system with SNAC prevents semaglutide from being broken down in the gastrointestinal tract. Additionally, the hydrophobic SNAC molecules make semaglutide more lipophilic, which enhances its transit into the bloodstream and transcellular uptake across the stomach membrane 89. Other peptide medications, including insulin and octreotide, that are currently in clinical trials, were additionally made possible by co-formulation using protease enzyme blockers and additional permeation-enhancing substances. Other methods, such as pulmonary consumption, epidermal distribution, and the use of implanted pumps, are now being researched. These methods include the creation of inhalable insulin and micro-implantable delivery systems for insulin pumps. In the upcoming years, we anticipate that these innovations will be used for additional peptide medications 90. Collectively, such advancements in formulation are expected to expand the therapeutic application of peptide-based medications, including next-generation anticancer peptides, by improving bioavailability, patient compliance, and overall treatment efficacy.

### 3.1. Challenges in Peptide Delivery

**Figure 3:**
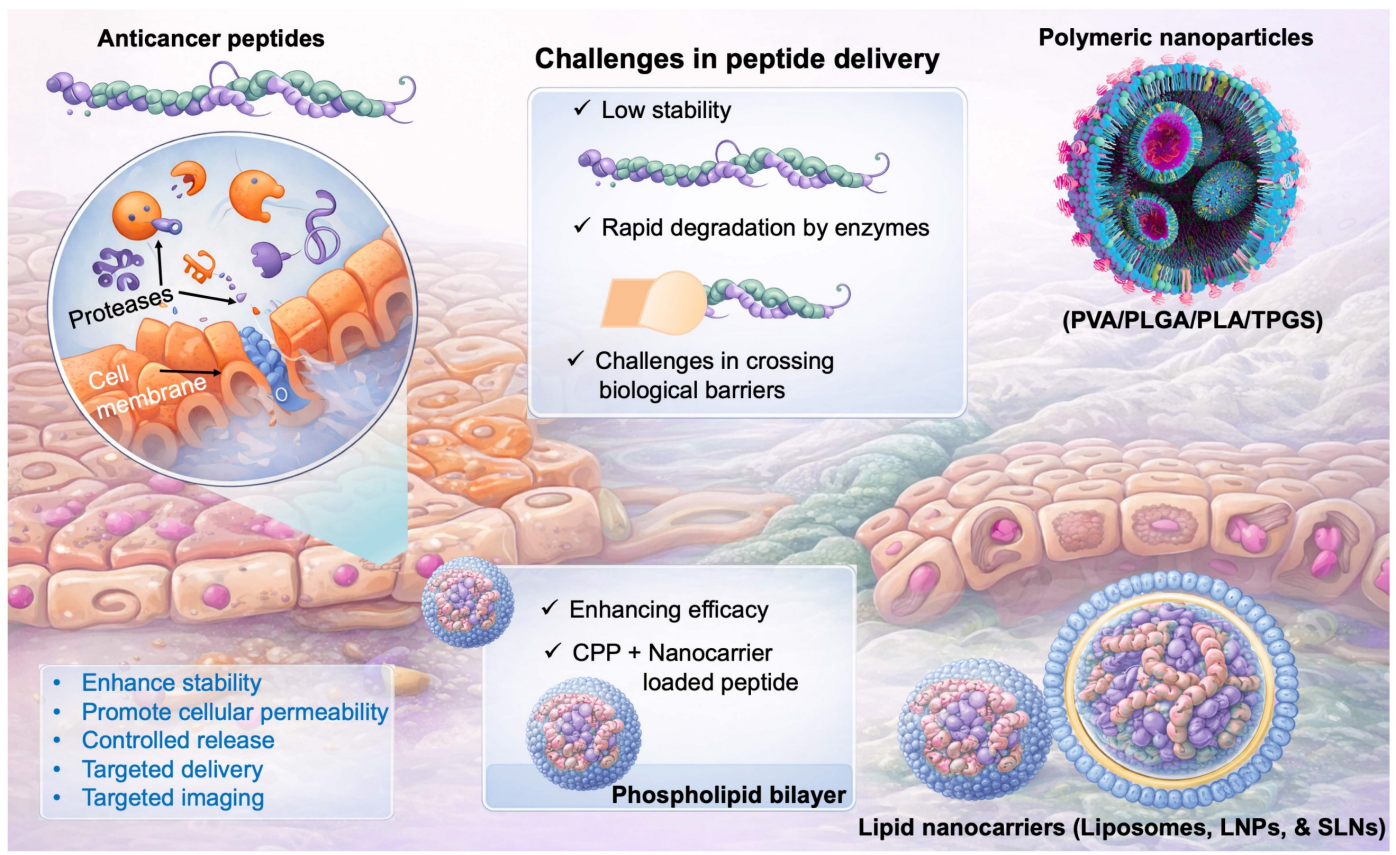
Graphical representation of challenges in peptide delivery to the cancer. ACPs face limitations such as instability, enzymatic degradation, and poor cellular uptake. Nanocarriers (polymeric and lipid-based) enhance stability, delivery, and targeting, improving therapeutic efficacy.

The clinical translation of ACPs is challenged by their intrinsic physicochemical limitations. The low stability, deterioration in different biological environments, and movement across different biological barriers are the key obstacles to the delivery of ACPs. Polymeric nanoparticulate vehicles are frequently utilised to increase the delivery of medication and extend protein/peptide drug absorption 91, 92. Given their stability in biological fluids, assisting prolonged drug release, and capacity to shield protein/peptide medications from degradation by enzymes, polymeric carriers offer significant benefits for drug administration. Polyesters, poly(ortho esters), polyphosphoesters, polyanhydrides, and polyelectrolytes are the most often utilised artificially produced polymers for drug administration. Because they are nontoxic, biocompatible, and biodegradable, poly(D, L-lactic acid) (PLA), poly(lactic-co-glycolic acid) (PLGA), and poly(vinyl alcohol) (PVA) are the most widely utilised polymeric materials for drug delivery. As a hydrophilic polymer, PVA dissolves in water when heated but is insoluble in many organic solvents. Numerous medications relying on proteins and peptides are soluble in water and hydrophilic that make it challenging to encapsulate with hydrophobic materials like PLGA or PLA. Consequently, PLA and PLGA-based copolymers containing a hydrophobic domain are frequently employed to boost the packaging of peptide/protein medications, although this requires additional processes during manufacturing 93. Collectively, overcoming stability issues, improving encapsulation efficiency, and achieving controlled release remain central themes in the development of effective delivery systems for next-generation anticancer peptides **(Fig. 3)**.

The FDA guidelines for peptide therapeutics, particularly for highly purified synthetic peptides referencing recombinant DNA (rDNA) origin drugs, emphasize stringent requirements in both IND-enabling studies and Chemistry, Manufacturing, and Controls (CMC). For CMC, the FDA requires a detailed description of the peptide's synthesis or isolation process, including its physicochemical properties, stability, and impurity control strategy. All drug substances and product specifications must ensure identity, strength, quality, and purity, with batch-to-batch consistency. Parenteral products should match the RLD in formulation and storage conditions 94. Furthermore, comprehensive stability studies must confirm that the peptide product maintains its chemical integrity, structural conformation, and biological activity under labelled storage conditions. Collectively, these requirements ensure that synthetic peptide therapeutics are safe, effective, and equivalent to their rDNA-derived counterparts 95.

Engineered peptide may have an immunogenicity risk that includes the intended immune activation and development of anti-drug antibodies that can reduce the therapeutic efficacy of the peptide. These kinds of responses may lead to alterations in the pharmacokinetic profile or adverse immune responses. The key is a strategy to eliminate this kind of problem with peptides, and improving the clinical translation can be done by sequence optimization, peptide modification and careful epitope screening 96.

#### 3.1.1. Pharmacokinetic and Regulatory Aspects of ACPs

In contrast to larger proteins or smaller-molecule drugs, peptides, defined as polymers of less than 50 amino acids with a molecular mass of ten or fewer kDa represent a rapidly expanding class of novel therapies with distinct pharmacokinetic properties. Natural peptides often have shorter plasma half-lives due to significant breakdown by proteolytic enzymes. Therapeutic peptides typically have a poor oral bioavailability due to their poor permeability and vulnerability to catabolic breakdown. They are given intravenously, subcutaneously, or intramuscularly, even though alternative methods like nasal delivery are also used 97, 98.

Numerous peptide-based medications have been licensed by the US Food and Drug Administration (FDA) as useful treatments for cancer. Peptides have an adequate safe profile, excellent specificity, permeability and targeted activation. They bind selectively to proteins and cell surface receptors, acting as either agonists or antagonists. They bind selectively to proteins and cell surface receptors, acting as either agonists or antagonists. They can be used as both diagnostic and therapeutic (theranostic) agents, and can be used as imaging substances for purposes of diagnosis. As a result, peptides are used in a variety of ways, including as payloads, linkers, and peptide conjugates 99.

### 3.2. Nanocarrier-based Delivery Systems

**Figure 4:**
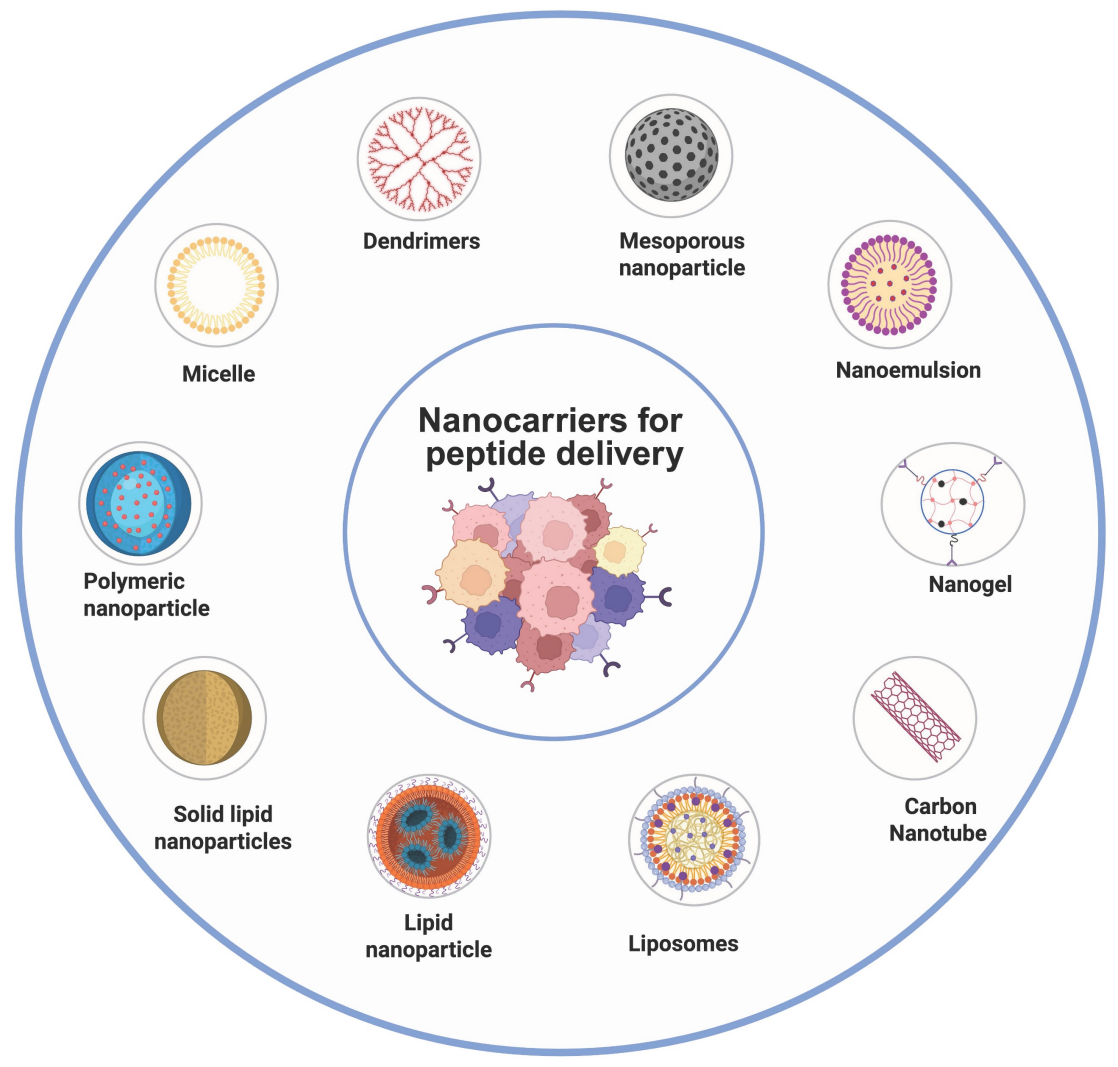
Schematic representation of the peptide-loaded nanocarriers used for cancer therapy. Various nanocarriers (e.g., liposomes, polymeric nanoparticles, micelles, dendrimers, and mesoporous silica) improve peptide stability, delivery, and targeting, enhancing therapeutic efficacy.

Nanoparticulate delivery systems are highly adaptable, submicron-sized drug delivery vehicles that are capable of transporting bioactive molecules with improved precision and efficiency. These systems include polymeric, lipidic, and inorganic nanoparticles, liposomes, and nanotubes. Drugs may be loaded inside the nanocarrier layers or scattered across the nanocarrier matrix. Since it is relatively easy to modify their shape, electrostatic charge, surface characteristics, and targeting ligands to control their cellular uptake, distribution in the body, selective targeting, and excretion, nanocarriers offer a number of benefits over conventional chemotherapy 100. The most promising treatment tool for malignancies and hereditary illnesses via peptide medications would significantly benefit from nanotechnology. The peptide-based nanocarrier system consists of two key components, that includes cancer targeting peptide and a therapeutic peptide(anticancer). Therapeutic peptides directly exert anticancer activity through various mechanisms, whereas targeting peptides primarily enhance delivery specificity by binding with tumor specific receptor without any cytotoxic effects. Accordingly, therapeutic peptides are designed for bioactivity and stability, while targeting peptides emphasise binding affinity and selectivity, influencing their distinct translational applications 101. In **Fig. 4,** we summarized nanosized lipid-based particles available for carrying peptide drugs.

#### 3.2.1. Lipid Nanocarriers

Lipid nanocarrier represents one of the most widely used platforms for the delivery of drugs. The lipid nanocarrier includes Liposomes, lipid nanoparticles (LNPs), and solid lipid nanoparticles (SLNs). Lipid nanoparticles deliver therapeutic agents by encapsulating hydrophilic drugs within aqueous cores and hydrophobic drugs within lipid bilayers or lipid matrices, and their cellular uptake occurs via membrane fusion or endocytosis and drug release is governed by lipid reorganisation or degradation.

##### 3.2.1.1 Liposomes for Peptide Delivery

The liposomes are the most frequently employed nanocarriers for a variety of hydrophobic and hydrophilic compounds because of their excellent biological compatibility with minimal immune activation, and the liposomes can be degraded naturally. Additionally, liposomes have been shown to improve chemotherapeutic drug solubility, regulate bio-distribution, and surface adaptation for specific and prolonged release. Liposomes have evolved over time from traditional and long-circulating formulations to targeted and immune-liposomes based systems. More recently, advances in composition and design have led to the development of stimuli-responsive and receptor targeted liposomes^102^, ^103^. Liposomes size, surface charge, lipid content, the number of lamellae, and surface modifications (with ligands or polymers) are adjustable during manufacturing, which regulate the durability of liposomes both *in vitro* and *in vivo*. These particles often have surface modifications to increase their circulatory and improve targeted delivery, as unmodified liposomes may be rapidly cleared by the mononuclear phagocyte system. At present, the FDA has authorised a number of liposomal-based drug delivery systems for the treatment of various illnesses, including cancer, viral infections, and fungal diseases; more of these liposomes have moved to advanced stages of clinical testing 104, 105.

In a study, Accardo *et al*., developed a new amphiphilic peptide derivative (*Mon*Y-BN) co-assembled 1,2-distearoyl-sn-glycero-3-phosphocholine (DSPC) liposomes loaded with doxorubicin (DOX) for targeted delivery to the bombesin receptor overexpressing cells. In mice that contained PC-3 xenografts, intravenous administration of DSPC/MonY-BN/Dox at a concentration of 10 mg/kg Dox resulted in a greater tumor suppression (60%) than nonspecific DSPC/DOX liposomes (36%), in comparison to control animals 106. In another study, Li *et al*., developed an iRGD peptide-modified phase transition liposome for hepatocellular imaging and therapy. The developed liposomes were loaded with 10-hydroxycamptothecin and indocyanine green to facilitate targeted tumor imaging and therapy. Further, *in vivo* studies demonstrated that targeted liposomes achieved tumor-specific accumulation, effective PA/ultrasound imaging, and significantly improved tumor suppression compared to non-targeted controls, suggesting superiority of the targeted therapy 107.

**Figure 5:**
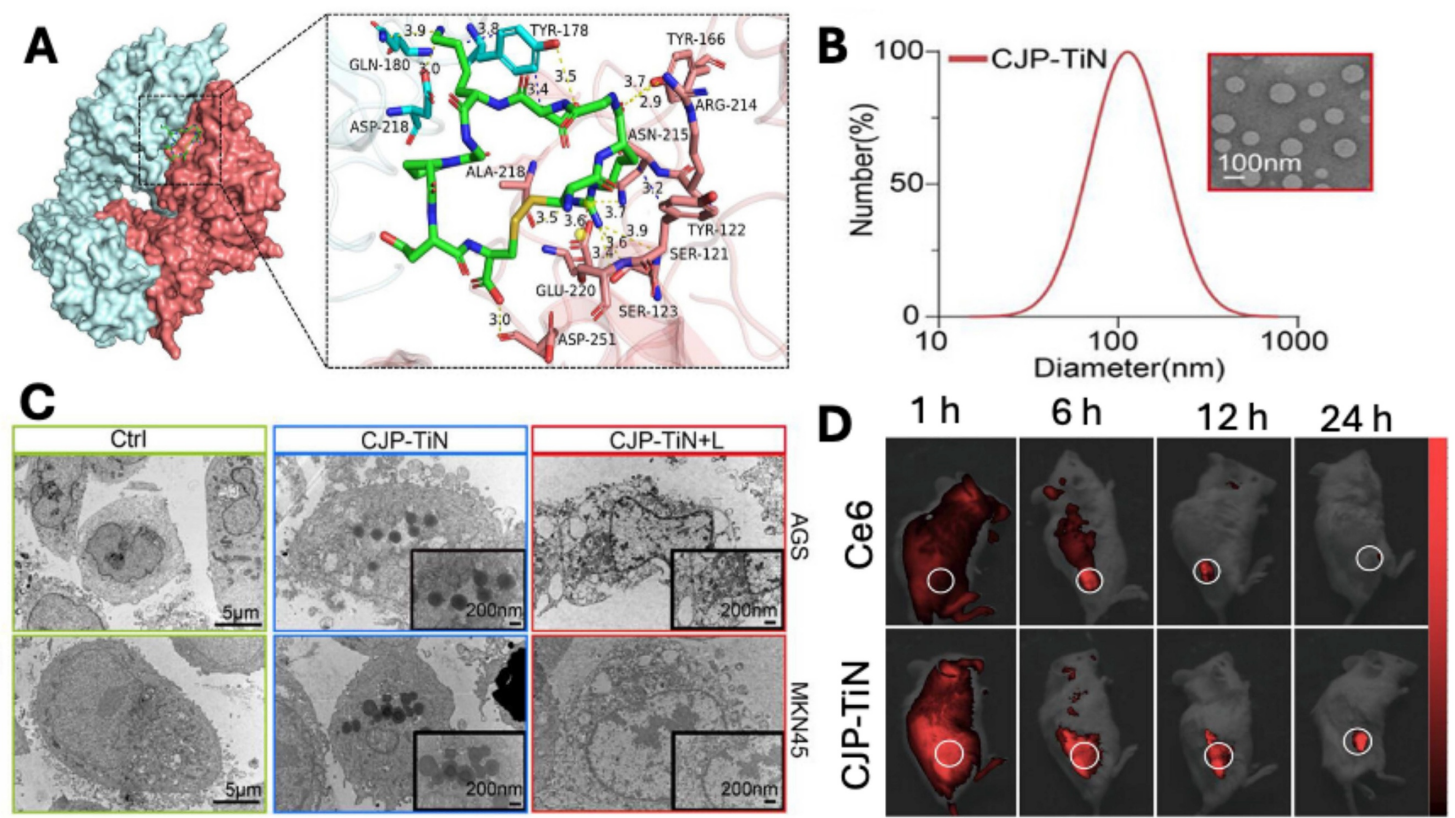
A) The molecular docking study between gastric carcinoma cell membrane receptor (αvβ3) and CJP-TiN ligand (iRGD). B) Particle size distribution and TEM image of liposomes (CJP-TiN). C) TEM images showing cellular uptake of liposomes in AGS & MKN45. D) *In vivo* imaging of MKN45-CDX mice following administration of free Ce6 and CJP-TiN liposomes at 1, 6, 12 and 24 hr. *Reproduced with permission from 108, Fig. 1, Fig. 3 & Fig. 5 (©2025 John Wiley & Sons)*.

Similarly, Ma *et al*. developed nanoliposomes (CJP-TiN) loaded with Chlorin e6(Ce6) and functionalised with Jolkinolide B, liposomes were of 100 nm size and were internalised via internalizing RGD peptide (iRGD) that induces ROS-caspase8/PANoptosis pathway leading to death of gastric cancer cells. The molecular docking study demonstrated a higher binding potential of RGD with αvβ3 was -8.2 kcal m-1 (**Fig. 5A**), suggesting a potential target in gastric cancer. The developed liposomes have uniform particle size distribution with a mean diameter of 109.36 ± 3.2 nm (**Fig. 5B**), which was essential for passive and receptor-mediated cellular uptake (**Fig. 5C**) in cancer cells, as demonstrated by TEM images. *In vivo* imaging demonstrated that liposomes were preferentially accumulated in the tumor site compared to the free Ce6 at 6 hr, following intravenous administration (**Fig. 5D**). In conclusion, the proposed CJP-TiN system efficiently targets tumor locations, triggers medication release through pH/ROS (reactive oxygen species) responsiveness, and enhances the synergistic benefits of the drug therapy by delivering Jolkinolide B and Chlorin e6 via functionalised liposomes in a stable manner 108.

Recently, Jiang *et al*., reported the development of a sphingomyelin (SM)-conjugated camptothecin (CPT) loaded liposomes functionalised with LinTT1 peptide(tumor-penetrating peptide) for targeted delivery to p32 overexpressed colon cancer. *Ex vivo* biodistribution study post 48 hr intravenous injection of free, non-targeted and targeted liposomes, demonstrating accumulation in tumor, liver and spleen. Targeted liposomes had higher localization in tumor compared to the non-targeted and free liposomes. Therapeutic efficacy study in HCT116 subcutaneous xenograft model, CPT (5 mg/kg), and Onivyde/Camptothesome (20 mg CPT/kg) were administered on the 6^th^ day, and tumor progression was monitored upto 33 days. Untreated group had rapid tumor growth, free CPT slowed down tumor growth and nontargeted liposomes had better inhibition of tumor growth compared to free drug, whereas targeted liposomes demonstrated superior inhibition of tumor growth and completely eradicated the tumor in one of five mice 109.

Taken together, liposomes are promising platforms for drug delivery, including proteins and peptides and have been translated into clinical practices. Presently, numerous studies based on liposomes have reported the usage of peptides as targeting moieties, yet very few have used loading peptides within the core, which could be due to the hydrophilic nature of peptides that limits their liposomal loading. The modification of peptide sequence would overcome the problem.

##### 3.2.1.2 Lipid Nanoparticles for Peptide Delivery

Another important subset of lipid-based NPs 110 is lipid nanoparticles (LNPs), which structurally resemble liposomes but differ in their internal organisation and are frequently utilised to deliver nucleic acids. Micelle formation creates the particle core, and the morphology may be changed depending on formulation and synthesis parameters. LNPs are primarily composed of four key components: phospholipids, cholesterol, cationic or ionizable lipids and PEGylated lipids. Phospholipids provide particle structure integrity, cholesterol enhances stability and membrane fusion, cationic or ionisable lipids bind negatively charged genetic material and facilitate endosomal escape, and PEGylated lipids improve stability and prolong circulation time 111, 112. Due to their facile manufacturing, small size, blood stability, and effective DNA delivery, LNPs are especially utilised for customised genetic drug delivery and treatment. Because ionisable LNPs have a nearly neutral charge at physiological pH but become charged in acidic endosomal compartments, this biological characteristic facilitates escaping from endosomal compartments during intracellular delivery. Although LNPs are widely recognized for nucleic acid delivery, their structural adaptability and tunable lipid composition also make them highly suitable for encapsulating and transporting peptide-based therapeutics. LNPs offer several advantages for peptide delivery that include enhancement of stability by protecting from enzymatic degradation, encapsulation within LNPs that shield from proteases, reducing premature clearance and prolonging systemic circulation 113, 114.

**Figure 6:**
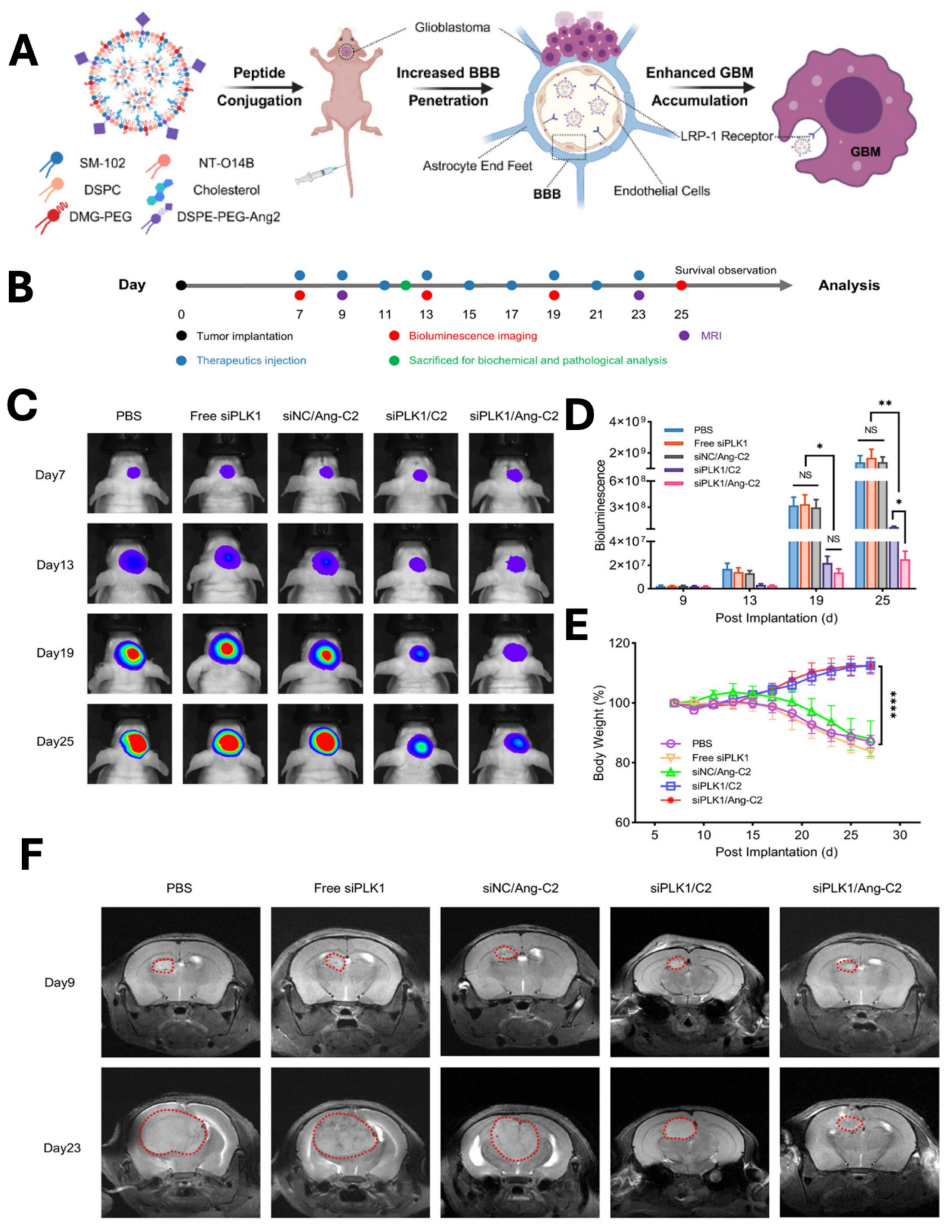
** A & B)** Schematic illustration of LNPs preparation, tumor implantation, imaging and treatment schedule for glioblastoma (GBM). *In vivo* **C)** bioluminescence imaging was performed following treatment with the control and nanoparticle groups, with quantitative analysis of **D)** bioluminescence signals and **E)** body weight measurements recorded at days 7, 13, 19, and 25. F) similarly CT imaging was performed at only 9 and 23 days. *Reproduced with permission from 115, Scheme 1, & Fig. 4 (©2025 American Chemical Society)*.

Glioblastoma multiforme (GBM) is the most prevalent malignant primary brain tumor and a WHO grade 4 glioma. Clinically managing GBM is very difficult, because drugs need to across the blood-brain barrier (BBB) and delivering efficient treatments to the brain is very challenging. Drug delivery with nanoparticles has shown great promise in treating this aggressive GBM. Tong *et al*., developed angiopep-2 peptide-modified LNPs for siRNA delivery to the brain **(Fig. 6A).** The developed LNPs depicted significant inhibition of GBM growth in the mouse model, with an enhancement in the 2.18-fold survival of the mice. *In vivo* bioluminescence imaging (**Fig. 6C**) revealed rapid tumor progression in the PBS-treated group, whereas Ang-C2 and C2 formulations markedly suppressed GBM growth compared with free siPLK1 and control nanoparticles. Quantitative analysis (**Fig. 6D**) confirmed significant reductions in tumor signal intensity in the Ang-C2 and C2 groups, demonstrating superior therapeutic efficacy. Throughout treatment, no significant changes in body weight were observed across groups (**Fig. 6E**), indicating favourable tolerability. Consistently, MRI imaging (**Fig. 6F**) further validated substantial tumor growth inhibition in mice treated with Ang-C2 and C2 formulations 115.

#### 3.2.1.3 Solid Lipid Nanoparticles for Peptide Delivery

Solid lipid nanoparticles are an effective and non-toxic substitute lipophilic colloidal carrier for drug delivery and are made from either physiological lipids or lipid molecules utilised as typical pharmaceutical additives. Unlike many polymer-based nanoparticle systems, SLN synthesis does not need the use of potentially hazardous organic solvents. This is in contrast to the majority of polymer-based microsphere and nanoparticle technologies. For therapeutic applications, proteins and antigens can be conjugated to SLN and administered either intravenously or via mucosal routes. By preventing proteolytic destruction upon delivery and releasing the protein in a regulated way, the matrix of lipids enhances the stability of peptides and proteins 116-118.

It has recently been shown that the octapeptide LSCQLYQR (LRp) inhibits the monomer-monomer interface of the human enzyme thymidylate synthase, therefore limiting the proliferation of ovarian cancer cells resistant to cis-platinum (cDDP) 119. The peptide needs the right delivery mechanism because it cannot pass through the cell membrane. To deliver this peptide, Sacchetti *et al*., developed LRp-loaded SLNs with a 150 nm particle size for the treatment of ovarian carcinoma. Notably, peptide-loaded SLNs significantly increased apoptosis compared with free peptide, which lacked cellular uptake, highlighting the importance of nanocarrier-mediated intracellular delivery. In contrast, incorporation of squalene destabilized peptide retention and accelerated drug release, reducing therapeutic efficacy. These findings underscore the potential of SLNs as a biocompatible platform for improving the intracellular delivery and anticancer activity of hydrophilic therapeutic peptides 120.

**Figure 7:**
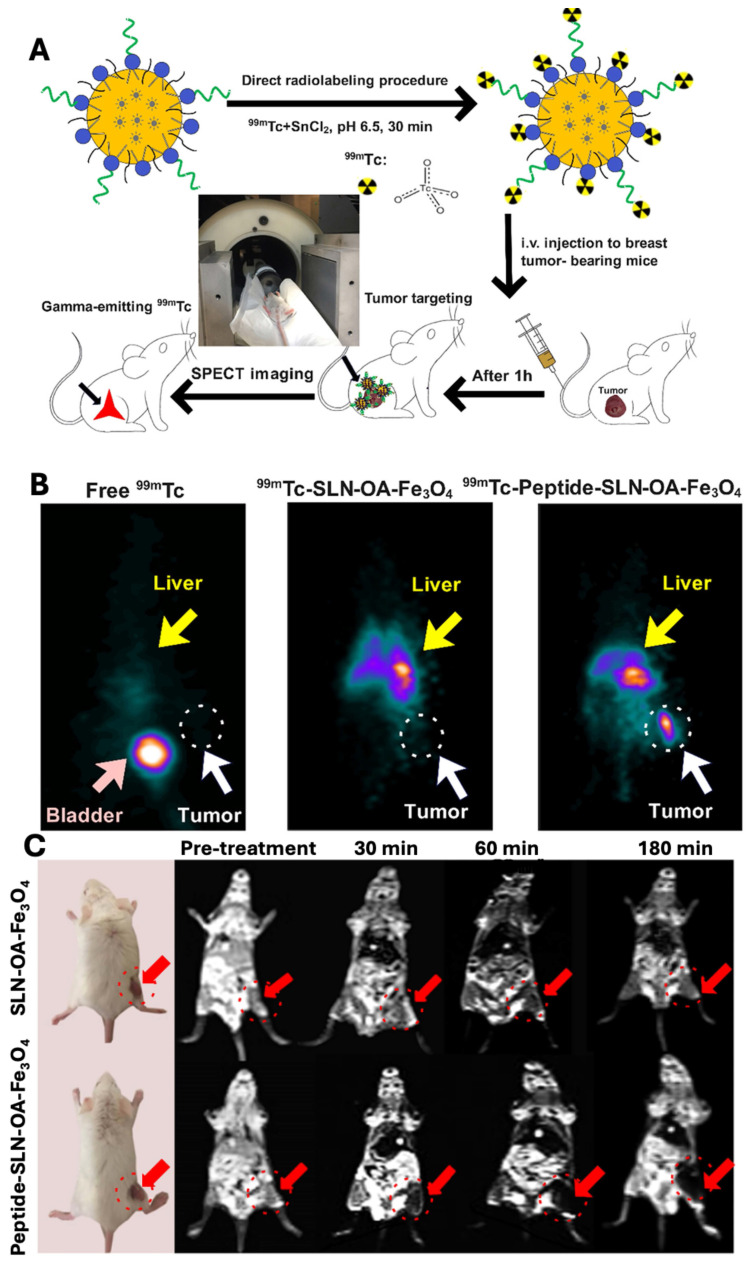
** A)** Schematic illustration of radiolabelling and *in vivo* SPECT imaging procedure. **B)** *In vivo* 3D SPECT imaging in the balb/c mice bearing 4T1 mammary carcinoma. **C)** *In vivo* MRI in the balb/c mice bearing 4T1 mammary carcinoma. *Reproduced with permission from 121, Fig. 5 & 6 (©2025 American Chemical Society).*

The invasive nature of triple-negative breast cancer (TNBC) and the absence of specific therapies make it a serious problem. Promising therapies for TNBC early diagnosis and monitoring include targeted nanoparticles and high-resolution imaging methods. In another study, Rahdari *et al*., developed SLNs conjugated with a short targeting peptide derived from endostatin for targeting of integrin αvβ3, overexpressed in TNBC. The developed SLNs was loaded with superparamagnetic iron oxide nanoparticles (SPIONs) and 99mTc for simultaneous magnetic resonance imaging (MRI) and single-photon emission computed tomography (SPECT) imaging. As demonstrated by receptor-binding tests in 4T1 cells utilising flow cytometry and MRI, the conjugation of C-peptide markedly improved the targeting effectiveness *in vitro*. Peptide-conjugated SLNs were shown to accumulate in tumor tissues in *in vivo* investigations utilising a 4T1 mouse model, improving tumor-specific localisation in SPECT imaging and offering improved contrast in MRI. Dual-modality imaging showed superior tumor visualization in both 3D SPECT (**Fig. 7**B) and MRI (**Fig. 7C**), following the radiolabeling and imaging protocol illustrated in **Fig. 7A**. Collectively, this peptide-conjugated SLN system represents a promising targeted nanotheranostic platform for improving TNBC diagnosis and tumor-specific imaging 121.

Despite their multiple advantages, lipid carriers also face several challenges that include formulation instability, limited drug loading for certain compounds, accelerated blood clearance upon repeated administration, and storage-related leakage issues. Currently, research is focused on next-generation ionizable lipid carriers, tissue-specific targeting beyond the liver, and minimising immune activation; these efforts position the lipid nanocarriers as the most translationally mature nano-delivery platform to date. Given their clinical maturity and regulatory acceptance, lipid nanocarriers remain among the most translationally advanced nano-delivery systems for peptide-based anticancer therapeutics 122, 123.

##### 3.2.2. Polymeric Nanoparticles and Polymeric Micelles for Peptide Delivery

Polymeric nanocarrier is a highly versatile platform for the delivery of therapeutic peptides that deliver drugs through encapsulation within biodegradable polymer matrices or self-assembled micellar cores, and release the payload via diffusion, polymer erosion, or stimulus-responsive degradation under pH or enzymatic conditions. Their key advantages include excellent structural stability, tuneable release kinetics, flexibility in surface functionalization, and suitability for long-term and combination therapies 124.

A micelle is a collection of scattered surfactant molecules that accumulate in a fluid colloidal. Typically, micelles are generally spherical, ellipsoidal, and cylinder-shaped. The form and size of the micelles were primarily influenced by temperature, pH, and the percentage. The hydrophobic ends of many surfactant compounds come together in a micelle to form an oil-like core with little water interaction. Micelles occur if the overall system temperature exceeds the point of critical micelle temperature and the amount of surfactant exceeds the critical micelle concentration. Poly(ethylene oxide)-block-poly(l-amino acid), poly(ethylene oxide)-block-poly(ester), and poly(ethylene oxide)-block-poly(propylene oxide)-block-poly(ethylene oxide) are examples of amphiphilic block copolymers that can produce polymeric micelles. Water-insoluble pharmacological molecules or enzymes can be trapped using poly(ethylene oxide)-block-poly(l-amino acid). The primary benefits are enhanced dispersion of hydrophobic pharmaceuticals, prolonged release of encapsulated compounds, biocompatibility, and unmodified biological function. Different anticancer peptides integrated into block copolymers by polymerisation, combined treatment, physical entrapment, and chemical coupling 125.

In a study, Tao A *et al*.. demonstrated the development of pH-responsive polymeric micelles capable of loading therapeutic drug through a combination of polyionic complexation and pH-cleavable covalent bonding using carboxydimethylmaleic anhydride. These micelles remained stable under physiological conditions (pH 7.4) but efficiently released functional proteins at mildly acidic pH (6.5), therefore, demonstrates prolonged blood circulation and preserved protein bioactivity, thereby suitable for targeted *in vivo* protein delivery 126. The past few years have seen a significant increase in interest in polymeric nanoparticles due to their modest size-related characteristics. The possibility for controlled discharge of loaded drug, the capacity to shield drugs and other compounds with biological functions from the environment are the benefits of using polymeric nanoparticles as drug carriers. Organic solvents are frequently utilised in the initial step to dissolve the polymer in the majority of methods that call for the use of premade polymers. The finished product must also be free of solvent residues. Methods focused on the polymerisation of monomers enable more efficient incorporation of chemicals into polymeric NPs in just one chemical step. The formulations are typically obtained as aqueous colloidal particles, irrespective of the manufacturing method used 127. Recently, Ghosh A *et al*., reported the development of cell-penetrating, protein-recognising polymeric nanoparticles fabricated by using dynamic covalent chemistry and in combination with a double molecular imprinting strategy. These water-soluble nanoparticles show high specificity and nanomolar binding affinity toward target proteins, effectively disrupt native protein-protein interactions, and readily enter cells, making them promising platforms for studying and modulating protein functions *in vitro* and with potential applications in anticancer peptide and protein delivery 128.

#### 3.2.3. Dendrimers as Nanocarriers for Peptide Delivery

**Figure 8:**
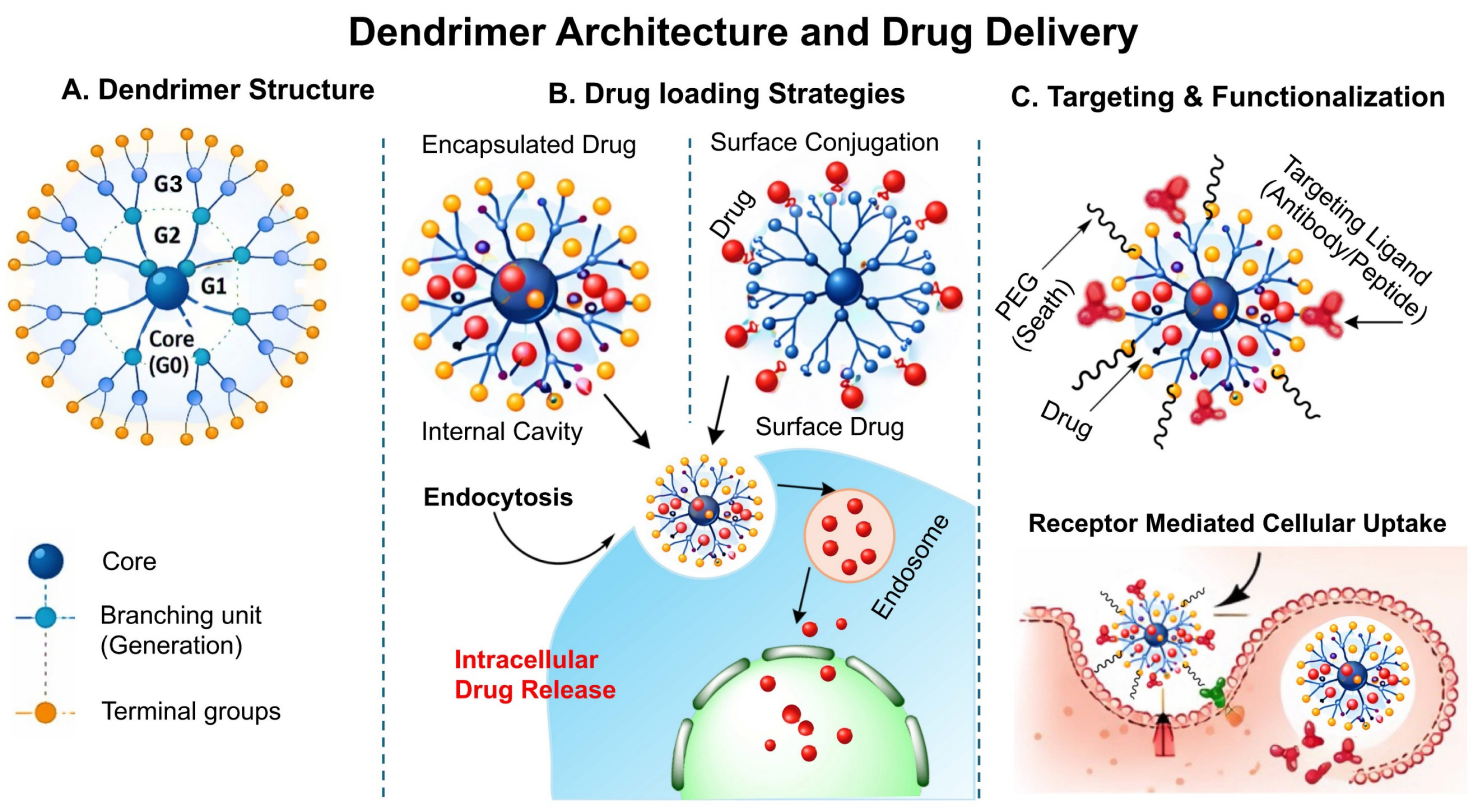
Schematic illustration of the dendrimer structure and drug delivery mechanism: A) Structural arrangement of dendrimer showing the central core, branching generation(G1-G3), their surface groups, B) Drug loading strategy in the dendrimer by encapsulation and surface conjugation, followed by endocytosis and intracellular release, C) Surface functionalization of dendrimer with PEG and targeting moiety for receptor mediated cellular uptake and controlled release of drug.

Dendrimers are highly branched, radially symmetric, nanoscale structures having a distinct, uniform, monodisperse shape that consists of an outermost shell, an inner shell, and a usually symmetrical core. Dendrimers act as nanocarriers by encapsulating drugs within internal cavities or by hydrophobic interactions, electrostatic force or alternatively covalent surface attachment, followed by endocytosis-mediated cellular uptake and intracellular drug release. Their highly defined architecture, monodispersed, high drug-loading efficiency, and multivalent surface functionality enable precise targeting and multifunctional applications. It is widely recognised that their three conventional macromolecular structural categories produce very multifaceted compositions with varying molecular weights. Each of the many types of dendrimers possesses biological characteristics, including being easily soluble, minimal cytotoxicity, being chemically stable, having electrostatic interaction, multiple valencies, and self-assemblage. Dendrimer structures start with a core, which is a central atom or collection of atoms. Through a range of chemical processes, the branches of additional atoms known as "dendrons" sprout from this core structure. Self-assembly is another exciting and quickly evolving field of chemistry. The exact, spontaneous joining of chemical species by particular, complementary intermolecular interactions is known as self- assembly. The self-assembling process of dendrite formations has garnered more attention lately 129. Schematic illustration of dendrimer structure, drug loading strategy and receptor mediated cellular uptake has been demonstrated in the **Fig. 8**.

In a study, Sun H et. al., reports the development of an anionic, phosphite-terminated phosphorus dendrimer-based nanocarrier (AK-137) that serves as a universal and bioactive platform for intracellular protein delivery. Unlike conventional cationic systems, AK-137 exhibits intrinsic anti-inflammatory activity and efficiently complexes diverse proteins, enabling effective cellular uptake, lysosomal escape, and preserved protein function. Notably, AK-137@fibronectin nanocomplexes show synergistic anti-inflammatory effects and robust therapeutic efficacy in acute lung injury and acute gout arthritis models by modulating NF-κB and PI3K/Akt signalling and promoting macrophage M2 polarisation, highlighting a safe and versatile strategy for protein-based anti-inflammatory therapy 130. In another study, Liu *et al*., developed a rationally designed boronic acid-rich dendrimer that enables highly efficient cytosolic delivery of native proteins with diverse sizes and isoelectric points while preserving their bioactivity. Notably, this system achieved robust intracellular delivery of Cas9 ribonucleoproteins and high-efficiency CRISPR-Cas9 genome editing in multiple cell lines, highlighting its broad potential as a general platform for protein, peptide and genome-editing therapeutics 131.

Although dendrimers are widely used as a carrier for drug delivery but suffer from low stability, especially when administered *in vivo*. To overcome these challenges, Yang *et al*., developed PAMAM dendrimer loaded with DOX (anticancer durg), ^64^CU (imaging agent) and functionalised with nucleolin (targeting peptide), which serves as a theranostic platform for treating and imaging of triple negative breast cancer model. The PET imaging in triple-negative tumor-bearing mice demonstrated that the targeted (^64^Cu-PAMAM-DOX-F3) dendrimer was accumulated significantly following 24 hr of injection compared to nontargeted (^64^Cu-PAMAM-DOX) dendrimer. They have very short circulation times and fairly comparable distribution characteristics in various organs and tissues. Furthermore, the effective delivery of DOX to MDA-MB-231 tumors by these PAMAM micelles was also demonstrated in *ex vivo* fluorescence imaging. These findings lead us to conclude that PAMAM-based micelles may be helpful in the targeted combinational therapy of cancer 132.

#### 3.2.4 Mesoporous Silica Nanoparticles (MSNs) for Peptide Delivery

Mesoporous silica nanoparticles deliver drugs by entrapping therapeutic agents within well-defined nanopores, followed by diffusion-controlled or stimulus-responsive release triggered by pH, enzymes, or redox conditions, with cellular uptake primarily via endocytosis. MSNs offer several advantages that include exceptionally high surface area, tunable pore size, excellent drug-loading capacity, and facile surface functionalization for targeting and imaging 133. However, concerns regarding slow biodegradation, long-term tissue accumulation, small pores hindering large proteins, premature leakage, protein denaturation, clearance (opsonisation), and silica-induced toxicity (cell lysis/immune response) limit their clinical progression. Currently, MSNs are widely explored as smart, stimuli-responsive and theranostic platforms in cancer and infectious disease research, but remain largely confined to preclinical studies due to unresolved safety and regulatory challenges 134.

### 3.3. Stimuli-Responsive and Targeted Delivery for Peptide Delivery

**Figure 9:**
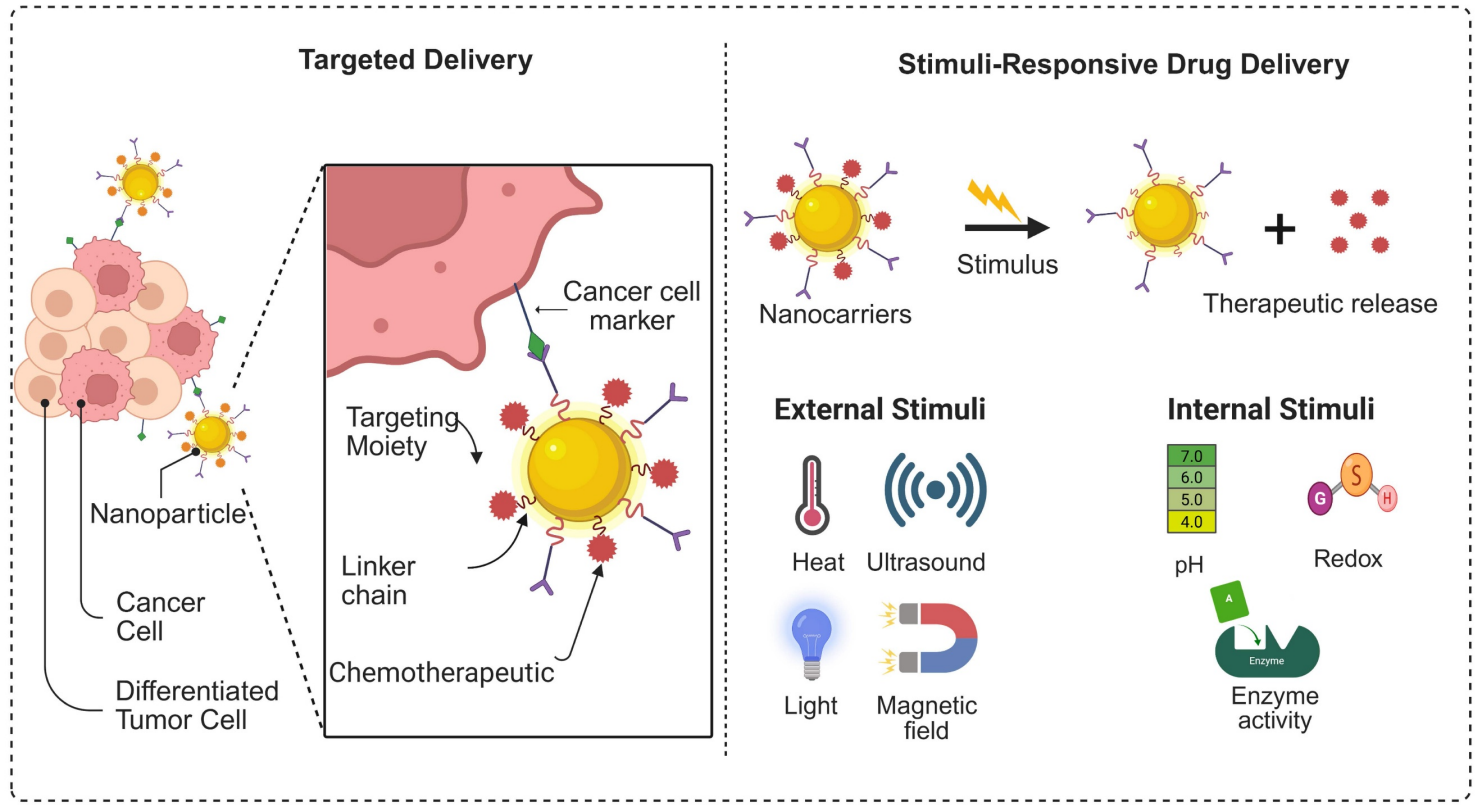
Graphical representation of the targeted and stimulus-responsive drug delivery system.

The goal of Nano-delivery is the tailoring of anticancer medicines to specific tumors without harming healthy cells. Through stimuli-responsive drug delivery systems, safe and efficient tumor-specific localisation has been made possible by recent developments in the field of onco-targeted therapy. Stimuli-responsive drug delivery systems possess additional benefits of improved bioavailability and targeted toxicity of tumor cells compared to traditional methods of drug delivery. Moreover, stimuli-responsive drug delivery technologies have improved chemotherapy-associated treatments due to their unique and beneficial features. Chemotherapy-related off-target events are lessened because stimulus-sensitive drug delivery systems prevent the release of cytotoxic medicines into healthy cells 135. Stimuli-responsive nanocarriers discharge their loaded drug when they sense specific changes in the environment. These changes occur in the body, such as differences in temperature, pH, redox potential, and the presence of certain enzymes. They can also respond to exogenous stimuli such as electromagnetic fields, light, radiation, and ultrasound. **Figure 9** illustrates a schematic representation of the construction of stimulus-responsive multifunctional nanocarriers and their potential drug delivery applications. Additionally, we have summarised the advantages and disadvantages of the nanocarriers used for peptide delivery in **Table 3**.

#### 3.3.1. pH-sensitive Systems as Nanocarrier for Peptide Delivery

Since many cancerous tissues have an acidic surrounding environment, pH-sensitive biomaterials for delivering drugs show a potential for targeted delivery and treatment. They could shield therapeutic molecules from metabolising and degrading throughout *in vivo* transportation and show pH-responsive discharge of the loaded drugs induced by the acidic microenvironment of diseased tissues, particularly for cancer therapy 136. Cancerous cells frequently favour an anaerobic process for the breakdown of glucose, which results in lactic acid being produced as a byproduct of inadequate oxidation. This is because cancerous cells are characterised by greater absorption of glucose to support their rapid growth and occasionally poor circulation for supplying enough oxygen. The "Warburg effect" is the result of elevated levels of lactic acid, lowering the pH of the tumor microenvironment. Consequently, when low-pH-responsive NPs come into contact with the acidic tumor environment during cancer therapy, the encapsulated peptide will be released 137.

Acidic breakage of linkage has been successfully used in the past few years to create pH-responsive polymers for both *in vitro* and *in vivo* uses 136. Common pH-sensitive nanocarriers used in cancer therapy include metal-organic frameworks (high loading and easy functionalization; possible framework instability), polymeric micelles (solubilize hydrophobic drugs with tunable release; dilution-driven instability), liposomes (biocompatible and clinically familiar; drug leakage and stability limits), gold nanoparticles (simple surface chemistry with imaging/therapy potential; non-biodegradable accumulation risk), dendrimers (high loading with controlled architecture; toxicity risk if strongly cationic), and mesoporous silica nanoparticles (cargo protection with controlled pore release; slow clearance and pore-size limits for large biomolecules), which collectively enable acid-triggered drug release in the tumor microenvironment. However, the system still face translation challenges related to stability, clearance, and safety 137. Ionizable membrane fusion liposomes have recently been developed to enable efficient transplantation of STING protein-enriched endoplasmic reticulum (ER) into tumor cells, thereby amplifying innate immune activation. This system utilises pH-responsive ionizable lipids to promote membrane fusion under acidic tumor microenvironment conditions, allowing cytosolic delivery of functional STING-cGAMP signalling complexes and robust induction of type I interferon and pro-inflammatory cytokines. The strategy represents a novel nanocarrier-mediated approach for tumor immunotherapy and induces long-term immune memory when combined with immune checkpoint blockade therapy 138.

Kirubanithy and Santhanam reported a biomass-derived, pH-responsive carbon quantum dot nanocarrier prepared from peanut shells (about 2.1 nm) and loaded with doxorubicin for tumor specific release. The DOX peanut shell carbon quantum dots showed enhanced drug release under acidic conditions (81.4% at pH 6.5 vs 55.4% at pH 7.4) with sustained release over 24 h and demonstrated strong in-vitro anticancer activity against MDA-MB-231 cells with efficient intracellular delivery (live/dead staining), supporting green-synthesised carbon dots as promising pH-triggered carriers for cancer therapy 139.

However, pH-responsive nanocarriers for peptide delivery face limitations like off-target delivery due to pH variations, instability in circulation, premature release before the target, difficulty achieving precise pH sensitivity (e.g., distinguishing subtle tumor pH differences), slow response times, and the complexity of the *in vivo* environment, impacting protein stability and release kinetics. Maintaining the nanocarrier's structural integrity while ensuring rapid, triggered protein release at the specific disease site remains a significant challenge 140.

#### 3.3.2. Light-Responsive Carrier for Peptide Delivery

The most effective light-responsive delivery systems use non-ionising radiation, are made of biodegradable materials, offer excellent spatial and temporal control over drug release, and are readily customisable to the intended clinical use. New delivery systems have been designed using a variety of light-based techniques, which can be categorised into three groups: photothermal, in which the energy from the absorbed photons is released through vibrational motion; photochemically triggered, in which the energy from the absorbed light is enough to spontaneously or through a photochemical reaction split covalent bonds; and photoisomerization, in which the surplus energy results in modifications to the structure 152. There is considerable promise for individualised and less invasive treatment options when multifunctional materials are combined into a single nanoplatform. Using multimodal synergistic processes, numerous studies demonstrated potential effectiveness in cancer combination treatment by presenting a simple yet adaptable approach to engineering a near-infrared (NIR) light-responsive nanomaterial 153.

**Figure 10:**
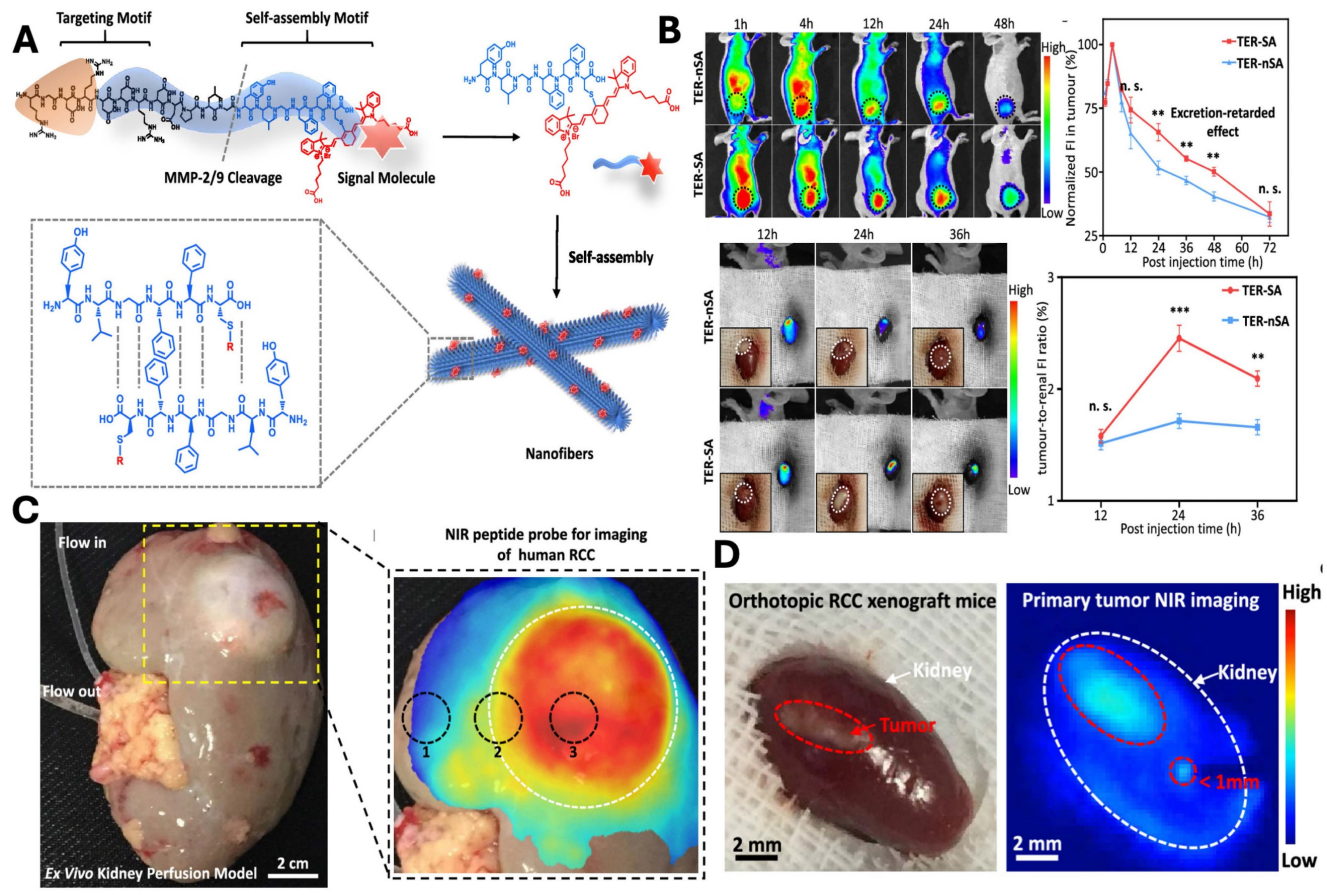
** A)** Schematic illustration and design of NIR peptide probe image-guided treatment of renal carcinoma. **B)** *In vivo* evaluation of the TER strategy showing prolonged tumor retention and optimal imaging window: representative NIR fluorescence images and quantitative analysis of TER-SA versus TER-nSA in subcutaneous and orthotopic 786-O RCC xenograft models, demonstrating enhanced tumor accumulation, delayed clearance, and peak tumor-to-kidney uptake ratio at 24 h post-injection. **C)** *Ex Vivo* kidney perfusion strategy and NIR peptide probe for tumor imaging. **D)** The TER strategy demonstrates complete tumor resection in the orthotopic model, demonstrating macroscopic image and its corresponding image post 24 hr of NIR fluorescence imaging. *Reproduced with permission from 154, Scheme 1, Fig. 3, Fig. 4 & 5 (©2020 American Chemical Society)*.

In a study, An *et al*., developed a tumor-specific excretion (TER) retarded NIR peptide probe to overcome challenges associated with imaging of metabolically active organs such as the kidney (**Fig. 10A**). The delivery system is composed of integrin-targeting RGD motif, an MMP-2/9-cleavable linker and self-assembly domains that enable the *in situ* assembly of the nanocarrier. Following the cleavage, the residual peptide promotes its self-assembly into β-sheet-rich nanofibers, which results in prolonged tumor retention and reduced renal clearance. *In vivo* study demonstrated enhanced tumor accumulation, reduced clearance, and optimum imaging window of 24 hr (**Fig. 10B**). The strategy was further validated in an *ex vivo* human kidney perfusion system, confirming its translational potential for clinical tumor identification (**Fig. 10C**). Finally, TER guided surgery enables precise visualisation and tumor resection with reduced postoperative recurrence in the orthotopic model (**Fig. 10D**) 154.

#### 3.3.3. Enzyme-Responsive Carriers for Peptide Delivery

Enzyme-responsive nano carriers represent a class of smart nano carriers that release the cargo in the presence of the specific enzyme and are located in a specific microenvironment. They are designed in such a way that they are inactive at normal microenvironment, but are activated in the presence of the targeted enzyme and release their payload. The functioning of the body is supported by enzymes, which are dysregulated in various illness-related environments and abnormal processes within cells. It is very promising to use changed enzyme production and function for therapeutic targeting, drug release, and diagnostics. Enzyme-responsive nanomaterials for controlled drug release have advanced significantly and are being investigated as an essential category of therapeutic delivery devices in nanotechnology when paired with the rapidly expanding field of biotechnology. Numerous enzymatic methods can be used to initiate the discharge of drugs. Through site-specific enzyme breaking down, therapeutics can be released from various nanocarriers. Drugs can be loaded into nanoparticles by binding through covalent bonds or through physical packaging using a caged porous material, self-assembling system, or cross-linked framework. Enzymes can also stimulate drug carriers, exposing the targeted ligand for internalisation into certain cells. Enzymes can also help produce specific goods, such as pH, which encourages the dissolution of drugs from transporters 155.

Hao *et al*. developed enzyme-responsive biomimetic ferritin nanoparticles (MMFn) that “deactivate” natural cytolytic peptides during circulation to minimise hemolysis, then reactivate them selectively in tumors via matrix metalloproteinase-2/9-cleavable linkers. MMFn showed strong tumor targeting and antitumor efficacy across multiple xenograft and patient-derived models, while also modulating tumor signalling and inducing robust antitumor immunity with no evident systemic toxicity, supporting a versatile enzyme-triggered peptide delivery strategy for resistant and immunologically “cold” tumors 156. However, enzyme-responsive nanocarriers for protein and peptide delivery face major hurdles, including scalable and reproducible manufacturing, and coping with enzyme heterogeneity (enzyme expression/activity varies across patients and even within the same tumor) 155, 157, 158.

#### 3.3.4. Ligand-Conjugated Nanocarrier for Peptide Delivery

Targeted nanocarrier systems based on ligand conjugation, such as antibodies, peptides, aptamers, folate, or integrin-binding motifs enable receptor-mediated uptake of peptide-loaded nanoparticles by cancer cells or tumor-associated immune cells. Ligand-conjugated delivery significantly improves intracellular peptide accumulation, enhances selectivity toward malignant tissues, and reduces off-target cytotoxicity, particularly in solid tumors with heterogeneous vasculature 159. Challenges for ligand-conjugated nanocarriers for protein/peptide delivery still face key challenges are preserving protein stability against enzymatic and pH-driven degradation, avoiding rapid immune recognition and clearance (opsonization and uptake by the reticuloendothelial system). Overcoming targeting barriers such as limited tissue penetration and the “binding-site barrier” is another challenging part 160.

In a study, Wang *et al.* leveraged ferritin as a natural ligand/targeting carrier for the transferrin receptor CD71, which they found to be highly expressed across multiple leukemia subtypes, to create As@Fn for receptor-mediated uptake. This ligand-guided nanomedicine markedly improved intracellular delivery of As(III) and reduced off-target exposure, producing strong anti-leukemia efficacy across cell lines and xenograft/PDX models and outperforming conventional arsenic therapy, supporting ligand-directed ferritin platforms for precision treatment of diverse hematologic malignancies 161.

**Figure 11:**
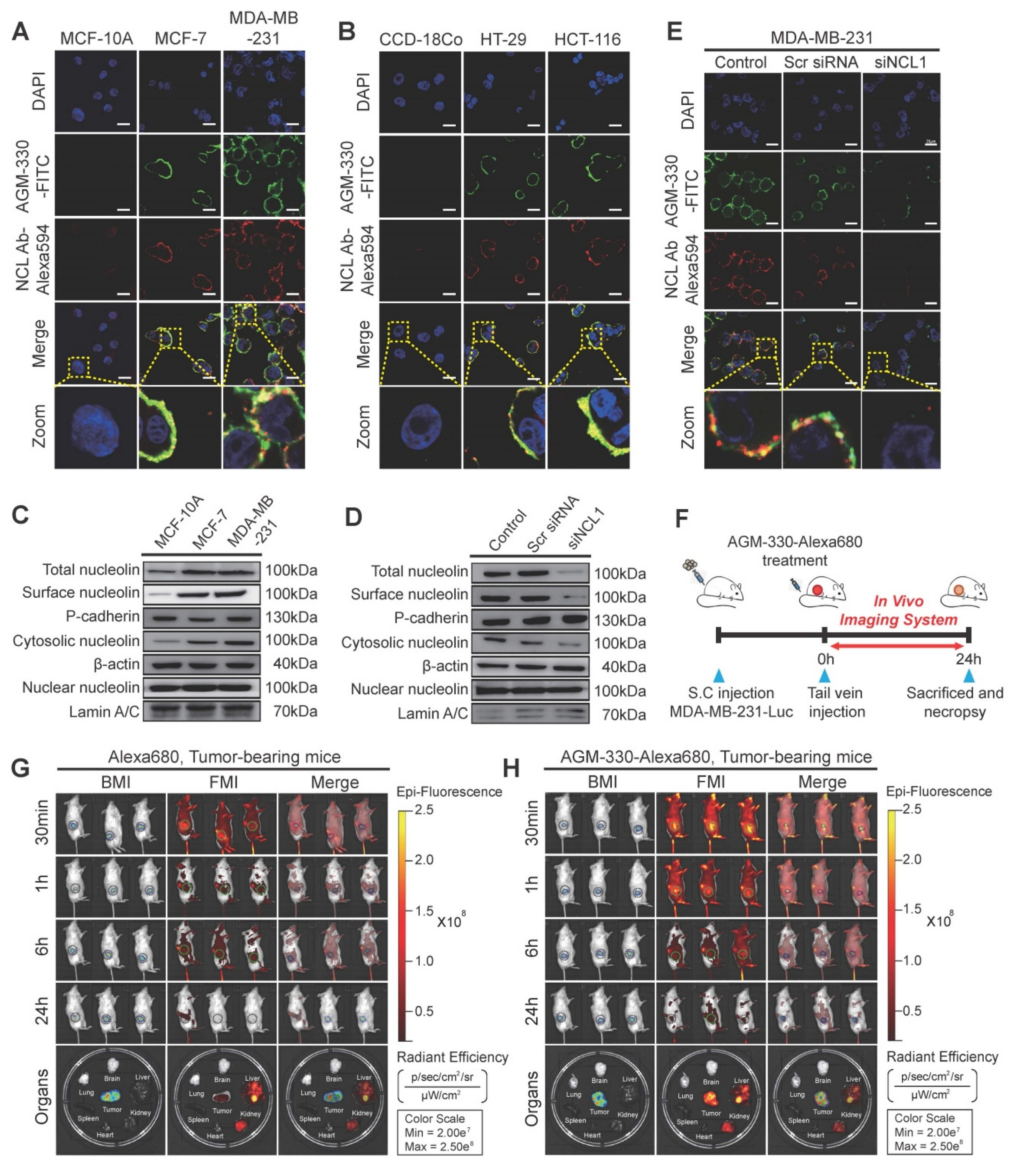
Binding Specificity of the AGM 330, *in vitro* and *in vivo*: **A & B)** confocal imaging of the cancer cell line (MCF-10A, MCF-, MDA-MB-231, HT-29, HCT-116) and normal cell line (CCD-18Co) incubated with 5 µmol/L AGM-330-FITC for 30 min at 37 °C. Nucleolin (NLC) was detected using an anti-NLC antibody by using Alexa Fluor 594-linked secondary antibody, and nuclei were stained with DAPI. MERGE shows co-localization of AGM-330-FITC, NCL, and nuclei. Scale bar, 20 µm. **C & D)** Subcellular localization of NCL with or without treatment of siNCL1 was analysed by immunoblotting of plasma membrane, cytosolic, and nuclear fractions. **E)** NCL knockdown reduces AGM-330-FITC membrane binding in MDA-MB-231 cells. **F)** Schematic of the *in vivo* tumor model and imaging protocol. **G, H)** *In vivo* fluorescence imaging of MDA-MB-231-luc tumor-bearing mice after intravenous injection of free Alexa680 or AGM-330-Alexa680. Representative whole-body and *ex vivo* organ images (heart, spleen, lung, brain, liver, kidney, and tumor) were acquired at indicated time points. *Reproduced with permission from 162, Fig. 3 (©2026 Ivyspring International Publisher)*.

A few years back, Kim *et al*. developed AGM 330 peptide that specifically binds to the nucleolin (NCL) receptor by one-bead-one-compound (OBOC) combinatorial method combined with a multiple-antigen-peptide (MAP) synthesis method. Further, they conjugated AGM330 to paclitaxel (PTX) to achieve targeted anticancer therapeutic efficacy. An *in vitro* study demonstrated that AGM 330-PTX conjugate was better than PTX alone. Further, the binding efficacy of the AGM 330 was validated by confocal fluorescence microscopy (**Fig. 11 A &B**), demonstrating selective binding with cancer cells and minimal binding with normal cells. Subcellular fractionation and immunoblot analyses revealed that nucleolin (NCL) was highly expressed on the plasma membrane of cancer cells, despite its classical localisation as a nuclear protein (**Fig. 11C-D**), whereas siRNA-mediated knockdown of NCL significantly reduced AGM-330 membrane binding (**Fig. 11E**). *In vivo* studies using a xenograft model demonstrated preferential tumor accumulation of AGM-330-fluorescein conjugate compared to free dye control. Further, AGM 330-PTX demonstrates significantly enhanced tumor efficacy compared to free PTX (**Fig. 11 F-H**). Collectively, these findings demonstrated that the dug peptide conjugate has better targetability and efficacy in the *in vitro* and *in vivo* models 162.

## 4. Future Perspectives and Challenges

ACPs are one of the promising next generation therapeutic platforms. Several ACPs derived from antimicrobial peptides have been emerging as a theranostic capable of serving as a diagnostic and therapeutic in a single platform. Their ability to target cancer cell membranes and disrupt tumor homeostasis provides an opportunity to circumvent classical receptor-mediated resistance mechanisms 163. Despite the advantages, several critical challenges must be addressed before ACPs translate into clinical applications. These include the expensive nature of production, their vulnerability to proteolytic cleavage, and short *in vivo* half-life. Addressing these limitations involves innovative peptide engineering strategies and advanced drug delivery systems. Sequences, secondary structures, net charges, amphipathicities, oligomerisation capacities, and high serum stabilities should all contribute to the success of ACPs 164. Importantly, the usage of ACPs may block several resistance processes as they are usually not targeted to particular intracellular or extrinsic receptors. This will ensure their continuous advancement in the therapeutic anti-cancer arsenal. Even if there are no established guidelines for the development of ACPs, a better understanding of the links between structures and activities, strengthened by creative chemical depictions and sophisticated mathematical methods, may offer useful resources for advancing ACPs from experimental molecules to a clinically viable theranostic system 9, 165. Emerging advances in ACP peptide research have been assisted by the AI-driven peptide discovery, leading to the rapid identification and optimisation of the peptide sequence with improved specificity and stability. Further integrating the ACPs with Immunotherapeutics, including checkpoint inhibitors and cancer vaccines, is expected to enhance therapeutic efficacy through synergistic immune activation. Moreover, the advancement of personalised peptide-based treatment tailored to individual tumor profile holds promise for improving precision oncology and clinical outcomes 166.

Future progress will surely rely on multidisciplinary convergence, leveraging artificial intelligence-assisted peptide design, precision nanofabrication, advanced biomarker-driven patient stratification, and harmonized regulatory frameworks. As the field transitions from empirical discovery to mechanism-driven and systems-engineered design, next-generation anticancer peptides are poised to move from promising experimental tools to clinically transformative platforms. With continued innovation in engineering, delivery, and translational strategy, ACPs based therapeutics have the potential to establish a new paradigm in precision oncology, one defined by selectivity, adaptability, and multifunctional therapeutic intelligence.

## Conclusion

ACPs are emerging therapeutics that are changing the landscape of oncology by integrating the molecular specificity, structural tunability, and multimodal activity within a single platform. Owing to therapeutic shortcomings, including drug resistance, systemic toxicity, and recurrence, the treatment continued to compromise the therapeutic outcomes. Although chemotherapy, radiotherapy, and surgery remain primary interventions for the treatment, their limitation seeks for the urgent demand of a selective, safe and durable therapeutic strategy. Conventional chemotherapy often has poor selectivity and contributes to the development of resistance, while surgery is effective at the early stage. Hence, advances in the rational design of peptides, their chemical modification, have significantly enhanced peptide stability, target selectivity, and pharmacokinetic and pharmacodynamic performance. When ACPs are combined with nanotechnology, evolved as programmable nanotheranotic systems capable of tumor targetability, controlled release, immunomodulation and simultaneous imaging of the tumor. Despite existing challenges, continued integration of computational design, nanotechnology, and translational strategies is expected to accelerate the development of ACP-based precision oncology.

## Figures and Tables

**Table 1:** List of anticancer peptides, with their source, molecular mechanism and use for cancer types

Peptides	Source	Molecular mechanism	Cancer types	Ref
Magainin II	Skin secretions of the African clawed frog, Xenopus Laevis	Membrane disruption, pore formation, apoptosis	Lungs, colon, breast, bladder	32, 43, 44
Melittin	Venom of honeybee	Membrane lysis, mitochondrial damage, apoptosis, NF-κB inhibition	Breast, liver, melanoma	45, 46
VGN50	Kaposi's sarcoma-associated herpesvirus (KSHV) transactivator protein (K-Rta)	block MYC-mediated gene transcription	Leukaemia, lymphoma	14
VGN73	KSHV- LANA protein	Targeting and cleaving the CHD4 protein	lymphoma	15
Cecropin A	Moth (Hyalophora cecropia)	Membrane disruption, pore formation, caspase mediated apoptosis	Leukaemia, bladder	47, 48
LL-37	Human cathelicidin	Mitochondrial damage, ROS generation, immune modulation	Lung, breast, ovarian	49-51
Buforin IIb	Histone H2A derivative	Apoptosis p53 pathway, DNA/RNA targeting	Prostate, liver	52, 53
LTX-315	Synthetic oncolytic peptide	ATP11b-PD-L1 targeting, immunological cell death	Pancreatic, breast	54, 55
ALRN-6924	Synthetic peptide	P53 reactivation	Lymphoma, solid tumor	56
SurVaxM	Survivin	Survivin specific cytotoxic T lymphocytes activation	Glioblastoma	57, 58
PR39	Porcine	Anti-angiogenic, pro-apoptotic	Colorectal	59
Tilapia Piscidin 4	Fish	ROS generation, membrane damage	Breast, glioblastoma	60, 61
Dermaseptin B2	Frog skin	Regulation of BAX/BBC3/AKT pathway	Breast	62
Temporin-1CEa	Frog skin	Membrane disruption	breast	63, 64
KT2	Synthetic	Membrane disruption & apoptosis	colon	65
Angiopep-2	Synthetic	LRP-1 targeting, enhances BBB permeation	glioblastoma	66, 67
DPX-Survivac	Survivin	Immune activation	Ovarian	68
UV1	Telomerase derived	Antitelomerase immunity induction	Lung, melanoma	69, 70
IMA901	Peptide vaccine	Immune activation	Renal cell carcinoma	71
DSP-7888	WT-1 antigen	CD4+/CD8+ T-cell activation	Leukaemia, solid tumor	72
NY-ESO-1	Cancer testis antigen	Cytotoxic T cells	Myeloma, melanoma	73
MAGE-A3	Tumor associated antigen	T cell activation	Multiple myeloma	74
K-α2 & K-α4	Viral FLIP protein of KSHV	disrupt FLIP-Atg3 interaction	lymphoma	13

**Table 2:** List of clinical trials registered in the clinical trials registry to evaluate the anti-cancer peptides therapeutic potential worldwide.

National Clinical Trial No.	Title	Peptides	Conditions	Phases	Status
04233476	99mTc-3PRGD2 SPECT/CT for integrin imaging of lung cancer (TRIIL)	c(RGDfK)2	Lung cancer metastatic	3	Completed
01479244	Efficacy and safety study of NeuVax™ (Nelipepimut-S or E75) vaccine to prevent breast cancer recurrence	E75 (HER2 369-377)	Breast cancer	3	Completed
00176046	Mistletoe extract in early or advanced breast cancer, a feasibility study	Viscotoxins	Metastatic breast cancer	4	Completed
02948309	Mistletoe therapy in primary and recurrent inoperable pancreatic cancer	Viscotoxins	Pancreatic cancer	3	Completed
00425360	Gemcitabine and capecitabine with or without vaccine therapy in treating patients with locally advanced or metastatic pancreatic cancer	GV1001	Pancreatic cancer	3	Completed
01401075	RCT With adjuvant mistletoe treatment in gastric cancer patients	Mistletoe lectins/Viscotoxins	Gastric cancer	4	Completed
01842165	^177^Lutetium-octreotate treatment prediction using multimodality imaging in refractory nets	DOTATATE (Tyr³-octreotate)	Gastroenteropancreatic neuroendocrine tumors	3	Completed
00094653	MDX-010 antibody, MDX-1379 melanoma vaccine, or MDX-010/MDX-1379 combination treatment for patients with unresectable or metastatic melanoma	IMDQVPFSV and YLEPGPVTA	Melanoma, metastases	3	Completed
01989572	Sargramostim, vaccine therapy, or sargramostim and vaccine therapy in preventing disease recurrence in patients with melanoma that has been removed by surgery	Tyrosinase peptide, gp100 peptide, and MART-1 peptide.	Iris melanoma, posterior uveal melanoma, mucosal melanoma	3	Completed
00090493	Study of MAGE-A3 and NY-ESO-1 immunotherapy in combo with DTPACE chemo and auto transplantation in multiple myeloma	MAGE-A3₁₆₈-₁₇₆ peptide & NY-ESO-1₁₅₆-₁₆₅ (C165V) peptide	Multiple myeloma	2, 3	Completed
00019682	Aldesleukin with or without vaccine therapy in treating patients with locally advanced or metastatic melanoma	gp100:209-217 (210M) melanoma peptide	Skin melanoma	3	Completed
00059475	Peptide vaccination for patients at high risk for recurrent melanoma	MART-1 & gp100: 209-217 (210M)	Melanoma	2	Completed
02264613	ALRN-6924 in patients with advanced solid tumors or lymphomas	ALRN-6924	Advanced solid tumors, lymphoma	1,2	Completed
02498665	A Study of DSP-7888 dosing emulsion in adult patients with advanced malignancies	DSP-7888	Myelodysplastic syndrome	2	Terminated
01265901	IMA901 in patients receiving sunitinib for advanced/metastatic renal cell carcinoma	IMA901	Renal cell carcinoma	3	Completed
01266083	WT1 vaccine treatment of patients in remission from acute myeloid leukemia (AML) or acute lymphoblastic leukemia	4 WT1-derived peptides	Acute myeloid leukemia	2	Completed
05163080	SurVaxM plus adjuvant temozolomide for newly diagnosed glioblastoma	Survivin peptide (SurVaxM)	Glioblastoma	2	Active
02785250	Study of DPX-survivac therapy in patients with recurrent ovarian cancer	Survivin peptide	Ovarian cancer	1,2	Completed
03311334	A Study of DSP-7888 dosing emulsion in combination with immune checkpoint inhibitors in adult patients with advanced solid tumors	DSP-7888	Solid tumors	1,2	Terminated

**Table 3:** Advantages and disadvantages of the nanocarriers used for peptide delivery

Drug Delivery System	Advantages	Limitations
*Lipid Based Nanocarrier System*		
Liposomes	Biocompatible, biodegradable, load hydrophilic and hydrophobic drug, surface modification possible, clinically validated	Drug leakage, limited stability, unmodified liposomes rapidly cleared by RES system 141, 142
Lipid Nanoparticles (LNPs)	Excellent for nucleic acid/ peptide delivery, ionizable lipid reduces toxicity, RES escape	Liver dominate accumulation, temperature sensitive storage 143
Solid Lipid Nanoparticles (SLNs)	Better stability, protects peptide degradation, controlled release	Poor drug loading, polymorphic transition, burst release possible 144
*Polymeric Nanocarriers and MSNs*		
Polymeric nanoparticles	Best for hydrophobic drug, surface functionalization possible, sustained release, self-assembly during preparation	Slow clearance, potential toxicity based on the polymer nature, complex synthesis process 127, 145
Polymeric Micelles	Excellent for hydrophobic drug, clinically viable, self-assembly ability to form micelles	Limited drug loading, premature drug release 146
Dendrimers	Better drug loading capacity, monodisperse system, surface functionalization feasible, enhanced targeting	Challenges in scale up, expensive, toxicity due to cationic dendrimers 147
Mesoporous silica nanoparticles (MSNs)	Higher surface area better contact, tunable pore size, better drug loading,	Non-biodegradable, long term toxicity 148
*Stimuli Responsive Nanocarrier System*		
pH responsive	Take advantages of acidic tumor microenvironment, pH targeted release (endosomes, lysosomes)	Heterogenicity in tumor pH, premature drug release 137
Light responsive	Spatial and temporal control; minimally invasive activation	Specially type of instrument requires for triggering the drug release, low light penetration (UV/Visible) 149
Enzyme responsive	Higher selectivity due to activation by specific tumor enzyme	Level of enzyme expression varies among patients 150
Ligand conjugated	Receptor mediated cellular uptake, higher specificity, improves tumor accumulation	Heterogenicity in the receptor expression, complexed optimization process 151

## References

[1] Siegel RL, Kratzer TB, Giaquinto AN, Sung H, Jemal A (2025). Cancer statistics, 2025. CA Cancer J Clin.

[2] Siegel RL, Kratzer TB, Wagle NS, Sung H, Jemal A (2026). Cancer statistics, 2026. CA Cancer J Clin.

[3] Maeda H, Khatami M (2018). Analyses of repeated failures in cancer therapy for solid tumors: poor tumor-selective drug delivery, low therapeutic efficacy and unsustainable costs. Clinical and translational medicine.

[4] Felício MR, Silva ON, Gonçalves S, Santos NC, Franco OL (2017). Peptides with Dual Antimicrobial and Anticancer Activities. Frontiers in chemistry.

[5] Varela-Quitián YF, Mendez-Rivera FE, Bernal-Estévez DA (2025). Cationic antimicrobial peptides: potential templates for anticancer agents. Frontiers in medicine.

[6] Sood A, Jothiswaran VV, Singh A, Sharma A (2024). Anticancer peptides as novel immunomodulatory therapeutic candidates for cancer treatment. Explor Target Antitumor Ther.

[7] Zhang Y, Wang C, Zhang W, Li X (2023). Bioactive peptides for anticancer therapies. Biomaterials translational.

[8] Manzari MT, Shamay Y, Kiguchi H, Rosen N, Scaltriti M, Heller DA (2021). Targeted drug delivery strategies for precision medicines. Nature reviews Materials.

[9] Chiangjong W, Chutipongtanate S, Hongeng S (2020). Anticancer peptide: Physicochemical property, functional aspect and trend in clinical application (Review). Int J Oncol.

[10] Baral KC, Choi KY (2025). Barriers and Strategies for Oral Peptide and Protein Therapeutics Delivery: Update on Clinical Advances. Pharmaceutics.

[11] Furman JL, Chiu M, Hunter MJ (2015). Early engineering approaches to improve peptide developability and manufacturability. The AAPS journal.

[12] Zhang D, Yuan Y, Zeng Q, Xiong J, Gan Y, Jiang K (2024). Plant protein-derived anti-breast cancer peptides: sources, therapeutic approaches, mechanisms, and nanoparticle design. Frontiers in pharmacology.

[13] Lee JS, Li Q, Lee JY, Lee SH, Jeong JH, Lee HR (2009). FLIP-mediated autophagy regulation in cell death control. Nat Cell Biol.

[14] Shimoda M, Lyu Y, Wang KH, Kumar A, Miura H, Meckler JF (2021). KSHV transactivator-derived small peptide traps coactivators to attenuate MYC and inhibits leukemia and lymphoma cell growth. Commun Biol.

[15] Miura H, Wang KH, Inagaki T, Chuang F, Shimoda M, Izumiya C (2024). A LANA peptide inhibits tumor growth by inducing CHD4 protein cleavage and triggers cell death. Cell Chem Biol.

[16] Wu CH, Liu IJ, Lu RM, Wu HC (2016). Advancement and applications of peptide phage display technology in biomedical science. J Biomed Sci.

[17] Molek P, Strukelj B, Bratkovic T (2011). Peptide phage display as a tool for drug discovery: targeting membrane receptors. Molecules (Basel, Switzerland).

[18] Yang PP, Li YJ, Cao Y, Zhang L, Wang JQ, Lai Z (2021). Rapid discovery of self-assembling peptides with one-bead one-compound peptide library. Nat Commun.

[19] Shinbara K, Liu W, van Neer RHP, Katoh T, Suga H (2020). Methodologies for Backbone Macrocyclic Peptide Synthesis Compatible With Screening Technologies. Front Chem.

[20] Lam KS, Lake D, Salmon SE, Smith J, Chen ML, Wade S (1996). A One-Bead One-Peptide Combinatorial Library Method for B-Cell Epitope Mapping. Methods.

[21] Roof RA, Sobczyk-Kojiro K, Turbiak AJ, Roman DL, Pogozheva ID, Blazer LL (2008). Novel peptide ligands of RGS4 from a focused one-bead, one-compound library. Chem Biol Drug Des.

[22] Wang DY, Wang L, Mi A, Wang J (2025). AI-Assisted Protein-Peptide Complex Prediction in a Practical Setting. J Comput Chem.

[23] Fatima I, Rehman A, Wang Z, Ur Rehman H, Aldaw M, Warraich DA (2026). AI-driven peptide discovery for endometrial cancer: deep generative modeling and molecular simulation in the big data era. J Comput Aided Mol Des.

[24] Cheng C, Cui H, Yu X, Li W (2025). Screening and Validation: AI-Aided Discovery of Dipeptidyl Peptidase-4 Inhibitory Peptides from Hydrolyzed Rice Proteins. Foods.

[25] Chen Z, Wang R, Guo J, Wang X (2024). The role and future prospects of artificial intelligence algorithms in peptide drug development. Biomed Pharmacother.

[26] Hashemi S, Vosough P, Taghizadeh S, Savardashtaki A (2024). Therapeutic peptide development revolutionized: Harnessing the power of artificial intelligence for drug discovery. Heliyon.

[27] Szymczak P, Zarzecki W, Wang J, Duan Y, Wang J, Coelho LP (2025). AI-Driven Antimicrobial Peptide Discovery: Mining and Generation. Acc Chem Res.

[28] Sharma K, Sharma KK, Sharma A, Jain R (2023). Peptide-based drug discovery: Current status and recent advances. Drug Discov Today.

[29] Anand U, Bandyopadhyay A, Jha NK, Pérez de la Lastra JM, Dey A (2023). Translational aspect in peptide drug discovery and development: An emerging therapeutic candidate. Biofactors.

[30] Muttenthaler M, King GF, Adams DJ, Alewood PF (2021). Trends in peptide drug discovery. Nat Rev Drug Discov.

[31] Xie M, Liu D, Yang Y (2020). Anti-cancer peptides: classification, mechanism of action, reconstruction and modification. Open Biol.

[32] Lehmann J, Retz M, Sidhu SS, Suttmann H, Sell M, Paulsen F (2006). Antitumor activity of the antimicrobial peptide magainin II against bladder cancer cell lines. Eur Urol.

[33] Pandey P, Khan F, Khan MA, Kumar R, Upadhyay TK (2023). An Updated Review Summarizing the Anticancer Efficacy of Melittin from Bee Venom in Several Models of Human Cancers. Nutrients.

[34] Jaber S, Nemska V, Iliev I, Ivanova E, Foteva T, Georgieva N (2021). Synthesis and Biological Studies on (KLAKLAK)(2)-NH(2) Analog Containing Unnatural Amino Acid β-Ala and Conjugates with Second Pharmacophore. Molecules.

[35] Valero JG, Sancey L, Kucharczak J, Guillemin Y, Gimenez D, Prudent J (2011). Bax-derived membrane-active peptides act as potent and direct inducers of apoptosis in cancer cells. J Cell Sci.

[36] Ogasawara M (2024). Wilms' tumor 1 -targeting cancer vaccine: Recent advancements and future perspectives. Hum Vaccin Immunother.

[37] Santhanam M, Babu V, Shteinfer-Kuzmine A, Pandey SK, Gheber L, Raday G (2026). Survivin/BIRC5-derived peptide disrupts survivin dimerization and cell division and induces multifaceted anti-cancer effects. Mol Ther Oncol.

[38] Zhou H, Ma Y, Liu F, Li B, Qiao D, Ren P (2023). Current advances in cancer vaccines targeting NY-ESO-1 for solid cancer treatment. Front Immunol.

[39] Nishida N, Yano H, Nishida T, Kamura T, Kojiro M (2006). Angiogenesis in cancer. Vasc Health Risk Manag.

[40] Cao Y, Xue L (2004). Angiostatin. Semin Thromb Hemost.

[41] Hamano Y, Kalluri R (2005). Tumstatin, the NC1 domain of alpha3 chain of type IV collagen, is an endogenous inhibitor of pathological angiogenesis and suppresses tumor growth. Biochem Biophys Res Commun.

[42] Reardon DA, Nabors LB, Stupp R, Mikkelsen T (2008). Cilengitide: an integrin-targeting arginine-glycine-aspartic acid peptide with promising activity for glioblastoma multiforme. Expert Opin Investig Drugs.

[43] Yang D, Zou R, Zhu Y, Liu B, Yao D, Jiang J (2014). Magainin II modified polydiacetylene micelles for cancer therapy. Nanoscale.

[44] Cannon M (1987). A family of wound healers. Nature.

[45] Prince G, Assi A, Aoude M, Kourie HR, Haddad F (2025). Melittin, A Potential Game-changer in the Fight Against Breast Cancer: A Systematic Review. Anticancer Agents Med Chem.

[46] Cui Z, Zhou Z, Sun Z, Duan J, Liu R, Qi C (2024). Melittin and phospholipase A2: Promising anti-cancer candidates from bee venom. Biomed Pharmacother.

[47] Sang M, Zhang J, Zhuge Q (2017). Selective cytotoxicity of the antibacterial peptide ABP-dHC-Cecropin A and its analog towards leukemia cells. Eur J Pharmacol.

[48] Suttmann H, Retz M, Paulsen F, Harder J, Zwergel U, Kamradt J (2008). Antimicrobial peptides of the Cecropin-family show potent antitumor activity against bladder cancer cells. BMC Urol.

[49] Kuroda K, Okumura K, Isogai H, Isogai E (2015). The Human Cathelicidin Antimicrobial Peptide LL-37 and Mimics are Potential Anticancer Drugs. Front Oncol.

[50] Ji P, Zhou Y, Yang Y, Wu J, Zhou H, Quan W (2019). Myeloid cell-derived LL-37 promotes lung cancer growth by activating Wnt/β-catenin signaling. Theranostics.

[51] Habes C, Weber G, Goupille C (2019). Sulfated Glycoaminoglycans and Proteoglycan Syndecan-4 Are Involved in Membrane Fixation of LL-37 and Its Pro-Migratory Effect in Breast Cancer Cells. Biomolecules.

[52] Han Y, Lu M, Zhou J (2019). Buforin IIb induces androgen-independent prostate cancer cells apoptosis though p53 pathway in vitro. Toxicon.

[53] Li D, Xu Y (2019). Buforin IIb induced cell cycle arrest in liver cancer. Anim Cells Syst (Seoul).

[54] Tang T, Huang X, Zhang G, Lu M, Hong Z, Wang M (2022). Oncolytic peptide LTX-315 induces anti-pancreatic cancer immunity by targeting the ATP11B-PD-L1 axis. J Immunother Cancer.

[55] Camilio KA, Wang MY, Mauseth B, Waagene S, Kvalheim G, Rekdal Ø (2019). Combining the oncolytic peptide LTX-315 with doxorubicin demonstrates therapeutic potential in a triple-negative breast cancer model. Breast Cancer Res.

[56] Guerlavais V, Sawyer TK, Carvajal L, Chang YS, Graves B, Ren JG (2023). Discovery of Sulanemadlin (ALRN-6924), the First Cell-Permeating, Stabilized α-Helical Peptide in Clinical Development. J Med Chem.

[57] Fenstermaker RA, Ciesielski MJ (2014). Challenges in the development of a survivin vaccine (SurVaxM) for malignant glioma. Expert Rev Vaccines.

[58] Ahluwalia MS, Reardon DA, Abad AP, Curry WT, Wong ET, Figel SA (2023). Phase IIa Study of SurVaxM Plus Adjuvant Temozolomide for Newly Diagnosed Glioblastoma. J Clin Oncol.

[59] Abaza MS, Bahman AM, Al-Attiyah RJ (2014). Valproic acid, an anti-epileptic drug and a histone deacetylase inhibitor, in combination with proteasome inhibitors exerts antiproliferative, pro-apoptotic and chemosensitizing effects in human colorectal cancer cells: underlying molecular mechanisms. Int J Mol Med.

[60] Su BC, Chen JY (2020). Pharmacological inhibition of p38 potentiates antimicrobial peptide TP4-induced cell death in glioblastoma cells. Mol Cell Biochem.

[61] Tajbakhsh A, Pasdar A, Rezaee M, Fazeli M, Soleimanpour S, Hassanian SM (2018). The current status and perspectives regarding the clinical implication of intracellular calcium in breast cancer. J Cell Physiol.

[62] Nasirabadi FK, Doosti A (2024). Dermaseptin B2 bioactive gene's potential for anticancer and anti-proliferative effect is linked to the regulation of the BAX/BBC3/AKT pathway. Med Oncol.

[63] Wang C, Zhou Y, Li S, Li H, Tian L, Wang H (2013). Anticancer mechanisms of temporin-1CEa, an amphipathic α-helical antimicrobial peptide, in Bcap-37 human breast cancer cells. Life Sci.

[64] Wang C, Tian LL, Li S, Li HB, Zhou Y, Wang H (2013). Rapid cytotoxicity of antimicrobial peptide tempoprin-1CEa in breast cancer cells through membrane destruction and intracellular calcium mechanism. PLoS One.

[65] Maraming P, Klaynongsruang S, Boonsiri P, Peng SF, Daduang S, Leelayuwat C (2019). The cationic cell-penetrating KT2 peptide promotes cell membrane defects and apoptosis with autophagy inhibition in human HCT 116 colon cancer cells. J Cell Physiol.

[66] Zhu Z, Zhai Y, Hao Y, Wang Q, Han F, Zheng W (2022). Specific anti-glioma targeted-delivery strategy of engineered small extracellular vesicles dual-functionalised by Angiopep-2 and TAT peptides. J Extracell Vesicles.

[67] Habib S, Singh M (2022). Angiopep-2-Modified Nanoparticles for Brain-Directed Delivery of Therapeutics: A Review. Polymers (Basel).

[68] Berinstein NL, Karkada M, Oza AM, Odunsi K, Villella JA, Nemunaitis JJ (2015). Survivin-targeted immunotherapy drives robust polyfunctional T cell generation and differentiation in advanced ovarian cancer patients. Oncoimmunology.

[69] Brunsvig PF, Guren TK, Nyakas M, Steinfeldt-Reisse CH, Rasch W, Kyte JA (2020). Long-Term Outcomes of a Phase I Study With UV1, a Second Generation Telomerase Based Vaccine, in Patients With Advanced Non-Small Cell Lung Cancer. Front Immunol.

[70] Lorigan P, Medina TM, Nyakas M, Rutten A, Feun LG, Cowey CL (2026). The telomerase vaccine UV1 combined with ipilimumab and nivolumab versus ipilimumab and nivolumab in advanced melanoma (INITIUM): A randomized open-label phase 2 study. Eur J Cancer.

[71] Rausch S, Kruck S, Stenzl A, Bedke J (2014). IMA901 for metastatic renal cell carcinoma in the context of new approaches to immunotherapy. Future Oncol.

[72] Suginobe N, Nakamura M, Takanashi Y, Ban H, Gotoh M (2023). Mechanism of action of DSP-7888 (adegramotide/nelatimotide) Emulsion, a peptide-based therapeutic cancer vaccine with the potential to turn up the heat on non-immunoreactive tumors. Clin Transl Oncol.

[73] Alsalloum A, Shevchenko JA, Sennikov S (2024). NY-ESO-1 antigen: A promising frontier in cancer immunotherapy. Clin Transl Med.

[74] Peled N, Oton AB, Hirsch FR, Bunn P (2009). MAGE A3 antigen-specific cancer immunotherapeutic. Immunotherapy.

[75] Malins LR (2018). Peptide modification and cyclization via transition-metal catalysis. Current opinion in chemical biology.

[76] Zhang P, Ma J, Yan Y, Chen B, Liu B, Jian C (2017). Arginine modification of lycosin-I to improve inhibitory activity against cancer cells. Org Biomol Chem.

[77] Hou D, Hu F, Mao Y, Yan L, Zhang Y, Zheng Z (2022). Cationic antimicrobial peptide NRC-03 induces oral squamous cell carcinoma cell apoptosis via CypD-mPTP axis-mediated mitochondrial oxidative stress. Redox Biol.

[78] Buchanan D, Mori S, Chadli A, Panda SS (2025). Natural Cyclic Peptides: Synthetic Strategies and Biomedical Applications. Biomedicines.

[79] Wu C, Wang H (2023). Recent Progress on Cyclic Peptides' Assembly and Biomedical Applications. Chembiochem.

[80] Martian PC, Tertis M, Leonte D, Hadade N, Cristea C, Crisan O (2025). Cyclic peptides: A powerful instrument for advancing biomedical nanotechnologies and drug development. J Pharm Biomed Anal.

[81] Song Q, Cheng Z, Kariuki M, Hall SCL, Hill SK, Rho JY (2021). Molecular Self-Assembly and Supramolecular Chemistry of Cyclic Peptides. Chem Rev.

[82] Veronese FM, Pasut G (2005). PEGylation, successful approach to drug delivery. Drug Discov Today.

[83] Hamley IW (2014). PEG-peptide conjugates. Biomacromolecules.

[84] Shoombuatong W, Schaduangrat N, Nantasenamat C (2018). Unraveling the bioactivity of anticancer peptides as deduced from machine learning. EXCLI journal.

[85] Huang YB, Wang XF, Wang HY, Liu Y, Chen Y (2011). Studies on mechanism of action of anticancer peptides by modulation of hydrophobicity within a defined structural framework. Molecular cancer therapeutics.

[86] Nasiri F, Atanaki FF, Behrouzi S, Kavousi K, Bagheri M (2021). CpACpP: In Silico Cell-Penetrating Anticancer Peptide Prediction Using a Novel Bioinformatics Framework. ACS Omega.

[87] Chen Y, Mant CT, Farmer SW, Hancock RE, Vasil ML, Hodges RS (2005). Rational design of alpha-helical antimicrobial peptides with enhanced activities and specificity/therapeutic index. J Biol Chem.

[88] Bruno BJ, Miller GD, Lim CS (2013). Basics and recent advances in peptide and protein drug delivery. Ther Deliv.

[89] Solis-Herrera C, Kane MP, Triplitt C (2024). Current Understanding of Sodium N-(8-[2-Hydroxylbenzoyl] Amino) Caprylate (SNAC) as an Absorption Enhancer: The Oral Semaglutide Experience. Clin Diabetes.

[90] Wang L, Wang N, Zhang W, Cheng X, Yan Z, Shao G (2022). Therapeutic peptides: current applications and future directions. Signal Transduct Target Ther.

[91] Visan AI, Cristescu R (2023). Polysaccharide-Based Coatings as Drug Delivery Systems. Pharmaceutics.

[92] Blanco E, Shen H, Ferrari M (2015). Principles of nanoparticle design for overcoming biological barriers to drug delivery. Nat Biotechnol.

[93] Swider E, Koshkina O, Tel J, Cruz LJ, de Vries IJM, Srinivas M (2018). Customizing poly(lactic-co-glycolic acid) particles for biomedical applications. Acta biomaterialia.

[94] Elsayed YY, Kühl T, Imhof D (2025). Regulatory Guidelines for the Analysis of Therapeutic Peptides and Proteins. Journal of peptide science: an official publication of the European Peptide Society.

[95] De Groot AS, Roberts BJ, Mattei A, Lelias S, Boyle C, Martin WD (2023). Immunogenicity risk assessment of synthetic peptide drugs and their impurities. Drug Discov Today.

[96] Achilleos K, Petrou C, Nicolaidou V, Sarigiannis Y (2025). Beyond Efficacy: Ensuring Safety in Peptide Therapeutics through Immunogenicity Assessment. J Pept Sci.

[97] Diao L, Meibohm B (2013). Pharmacokinetics and pharmacokinetic-pharmacodynamic correlations of therapeutic peptides. Clin Pharmacokinet.

[98] Lebleu B (2024). Feature Collection in Peptide Therapeutics: Current Applications and Future Directions. Biomedicines.

[99] Al Musaimi O (2024). Peptide Therapeutics: Unveiling the Potential against Cancer-A Journey through 1989. Cancers (Basel).

[100] Alshawwa SZ, Kassem AA, Farid RM, Mostafa SK, Labib GS (2022). Nanocarrier Drug Delivery Systems: Characterization, Limitations, Future Perspectives and Implementation of Artificial Intelligence. Pharmaceutics.

[101] Wang Y, Zhang L, Liu C, Luo Y, Chen D (2024). Peptide-Mediated Nanocarriers for Targeted Drug Delivery: Developments and Strategies. Pharmaceutics.

[102] Large DE, Abdelmessih RG, Fink EA, Auguste DT (2021). Liposome composition in drug delivery design, synthesis, characterization, and clinical application. Adv Drug Deliv Rev.

[103] Zylberberg C, Matosevic S (2017). Bioengineered liposome-scaffold composites as therapeutic delivery systems. Ther Deliv.

[104] Nsairat H, Khater D, Sayed U, Odeh F, Al Bawab A, Alshaer W (2022). Liposomes: structure, composition, types, and clinical applications. Heliyon.

[105] Swaminathan J, Ehrhardt C (2012). Liposomal delivery of proteins and peptides. Expert Opin Drug Deliv.

[106] Accardo A, Salsano G, Morisco A, Aurilio M, Parisi A, Maione F (2012). Peptide-modified liposomes for selective targeting of bombesin receptors overexpressed by cancer cells: a potential theranostic agent. Int J Nanomedicine.

[107] Li H, Shi S, Wu M, Shen W, Ren J, Mei Z (2021). iRGD Peptide-Mediated Liposomal Nanoparticles with Photoacoustic/Ultrasound Dual-Modality Imaging for Precision Theranostics Against Hepatocellular Carcinoma. Int J Nanomedicine.

[108] Ma C, Gao L, Song K, Gu B, Wang B, Yu Y (2025). Targeted Dual-Responsive Liposomes Co-Deliver Jolkinolide B and Ce6 to Synergistically Enhance the Photodynamic/Immunotherapy Efficacy in Gastric Cancer through the PANoptosis Pathway. Adv Sci (Weinh).

[109] Jiang Y, Wang Z, Li W, Ma T, Li M, Wu S (2025). Enhanced delivery of camptothecin to colorectal carcinoma using a tumor-penetrating peptide targeting p32. Acta Biomater.

[110] Hald Albertsen C, Kulkarni JA, Witzigmann D, Lind M, Petersson K, Simonsen JB (2022). The role of lipid components in lipid nanoparticles for vaccines and gene therapy. Adv Drug Deliv Rev.

[111] Kaur N, Gautam P, Nanda D, Meena AS, Shanavas A, Prasad R (2024). Lipid Nanoparticles for Brain Tumor Theranostics: Challenges and Status. Bioconjug Chem.

[112] Gangavarapu A, Tapia-Lopez LV, Sarkar B, Pena-Zacarias J, Badruddoza AZM, Nurunnabi M (2024). Lipid nanoparticles for enhancing oral bioavailability. Nanoscale.

[113] El Moukhtari SH, Garbayo E, Amundarain A, Pascual-Gil S, Carrasco-León A, Prosper F (2023). Lipid nanoparticles for siRNA delivery in cancer treatment. J Control Release.

[114] Serpico L, Zhu Y, Maia RF, Sumedha S, Shahbazi MA, Santos HA (2024). Lipid nanoparticles-based RNA therapies for breast cancer treatment. Drug Deliv Transl Res.

[115] Tong H, Ma Z, Yu J, Li D, Zhu Q, Shi H (2025). Optimizing Peptide-Conjugated Lipid Nanoparticles for Efficient siRNA Delivery across the Blood-Brain Barrier and Treatment of Glioblastoma Multiforme. ACS Chem Biol.

[116] António JA, Eliana S (2007). Solid lipid nanoparticles as a drug delivery system for peptides and proteins. Advanced Drug Delivery Reviews.

[117] Gamboa L, Zamat AH, Thiveaud CA, Lee HJ, Kulaksizoglu E, Zha Z (2025). Sensitizing solid tumors to CAR-mediated cytotoxicity by lipid nanoparticle delivery of synthetic antigens. Nat Cancer.

[118] Pandey S, Shaikh F, Gupta A, Tripathi P, Yadav JS (2022). A Recent Update: Solid Lipid Nanoparticles for Effective Drug Delivery. Adv Pharm Bull.

[119] Cannazza G, Cazzato AS, Marraccini C, Pavesi G, Pirondi S, Guerrini R (2014). Internalization and stability of a thymidylate synthase Peptide inhibitor in ovarian cancer cells. J Med Chem.

[120] Sacchetti F, Marraccini C, D'Arca D, Pelà M, Pinetti D, Maretti E (2015). Enhanced anti-hyperproliferative activity of human thymidylate synthase inhibitor peptide by solid lipid nanoparticle delivery. Colloids Surf B Biointerfaces.

[121] Rahdari T, Ghafouri H, Ramezanpour S, Ardestani MS, Asghari SM (2025). Design and Characterization of Peptide-Conjugated Solid Lipid Nanoparticles for Targeted MRI and SPECT Imaging of Breast Tumors. ACS Omega.

[122] Khan S, Sharma A, Jain V (2023). An Overview of Nanostructured Lipid Carriers and its Application in Drug Delivery through Different Routes. Adv Pharm Bull.

[123] Wang J, Ding Y, Chong K, Cui M, Cao Z, Tang C (2024). Recent Advances in Lipid Nanoparticles and Their Safety Concerns for mRNA Delivery. Vaccines (Basel).

[124] De R, Mahata MK, Kim KT (2022). Structure-Based Varieties of Polymeric Nanocarriers and Influences of Their Physicochemical Properties on Drug Delivery Profiles. Adv Sci (Weinh).

[125] Hanafy NAN, El-Kemary M, Leporatti S (2018). Micelles Structure Development as a Strategy to Improve Smart Cancer Therapy. Cancers.

[126] Tao A, Huang GL, Igarashi K, Hong T, Liao S, Stellacci F (2020). Polymeric Micelles Loading Proteins through Concurrent Ion Complexation and pH-Cleavable Covalent Bonding for In Vivo Delivery. Macromolecular Bioscience.

[127] Zielińska A, Carreiró F, Oliveira AM, Neves A, Pires B, Venkatesh DN (2020). Polymeric Nanoparticles: Production, Characterization, Toxicology and Ecotoxicology. Molecules.

[128] Ghosh A, Sharma M, Zhao Y (2024). Cell-penetrating protein-recognizing polymeric nanoparticles through dynamic covalent chemistry and double imprinting. Nature Communications.

[129] Abbasi E, Aval SF, Akbarzadeh A, Milani M, Nasrabadi HT, Joo SW (2014). Dendrimers: synthesis, applications, and properties. Nanoscale research letters.

[130] Sun H, Zhan M, Karpus A, Zou Y, Li J, Mignani S (2024). Bioactive Phosphorus Dendrimers as a Universal Protein Delivery System for Enhanced Anti-inflammation Therapy. ACS Nano.

[131] Liu C, Wan T, Wang H, Zhang S, Ping Y, Cheng Y (2019). A boronic acid-rich dendrimer with robust and unprecedented efficiency for cytosolic protein delivery and CRISPR-Cas9 gene editing. Science Advances.

[132] Yang J, Lu W, Xiao J, Zong Q, Xu H, Yin Y (2018). A positron emission tomography image-guidable unimolecular micelle nanoplatform for cancer theranostic applications. Acta Biomater.

[133] Vallet-Regí M, Colilla M, Izquierdo-Barba I, Manzano M (2017). Mesoporous Silica Nanoparticles for Drug Delivery: Current Insights. Molecules (Basel, Switzerland).

[134] Anas Al Tahan M, Marwah M, El-Zein H, Al Tahan S, Sanchez-Aranguren L (2025). Exploring mesoporous silica microparticles in pharmaceutical sciences: Drug delivery and therapeutic insights. International Journal of Pharmaceutics.

[135] Rahim MA, Jan N, Khan S, Shah H, Madni A, Khan A (2021). Recent Advancements in Stimuli Responsive Drug Delivery Platforms for Active and Passive Cancer Targeting. Cancers.

[136] Zhuo S, Zhang F, Yu J, Zhang X, Yang G, Liu X (2020). pH-Sensitive Biomaterials for Drug Delivery. Molecules.

[137] AlSawaftah NM, Awad NS, Pitt WG, Husseini GA (2022). pH-Responsive Nanocarriers in Cancer Therapy. Polymers (Basel).

[138] Yuan P, Yan X, Zhu T, Zong X, Chen X, Yang C (2025). Efficient transplantation of STING protein-enriched endoplasmic reticulum for tumor immunotherapy via ionizable membrane fusion liposome. Chemical Engineering Journal.

[139] Kirubanithy K, Santhanam A (2025). A pH-responsive nanocarrier of peanut shell carbon quantum dots as a promising delivery of doxorubicin for cancer therapy. Scientific Reports.

[140] Lu Y, Sun W, Gu Z (2014). Stimuli-responsive nanomaterials for therapeutic protein delivery. Journal of controlled release: official journal of the Controlled Release Society.

[141] Sercombe L, Veerati T, Moheimani F, Wu SY, Sood AK, Hua S (2015). Advances and Challenges of Liposome Assisted Drug Delivery. Front Pharmacol.

[142] Liu P, Chen G, Zhang J (2022). A Review of Liposomes as a Drug Delivery System: Current Status of Approved Products, Regulatory Environments, and Future Perspectives. Molecules.

[143] Jung HN, Lee SY, Lee S, Youn H, Im HJ (2022). Lipid nanoparticles for delivery of RNA therapeutics: Current status and the role of in vivo imaging. Theranostics.

[144] M NK, S S, P SR, Narayanasamy D (2024). The Science of Solid Lipid Nanoparticles: From Fundamentals to Applications. Cureus.

[145] Eltaib L (2025). Polymeric Nanoparticles in Targeted Drug Delivery: Unveiling the Impact of Polymer Characterization and Fabrication. Polymers (Basel).

[146] Turnovská A, Etrych T (2025). Polymeric micelles in advanced photodynamic therapy: Design, delivery and translational prospects. Int J Pharm X.

[147] Palmerston Mendes L, Pan J, Torchilin VP (2017). Dendrimers as Nanocarriers for Nucleic Acid and Drug Delivery in Cancer Therapy. Molecules.

[148] Narayan R, Nayak UY, Raichur AM, Garg S (2018). Mesoporous Silica Nanoparticles: A Comprehensive Review on Synthesis and Recent Advances. Pharmaceutics.

[149] Su S, P MK (2020). Recent Advances in Nanocarrier-Assisted Therapeutics Delivery Systems. Pharmaceutics.

[150] Gressler S, Hipfinger C, Part F, Pavlicek A, Zafiu C, Giese B (2025). A systematic review of nanocarriers used in medicine and beyond - definition and categorization framework. J Nanobiotechnology.

[151] Yan S, Na J, Liu X, Wu P (2024). Different Targeting Ligands-Mediated Drug Delivery Systems for Tumor Therapy. Pharmaceutics.

[152] Linsley CS, Wu BM (2017). Recent advances in light-responsive on-demand drug-delivery systems. Ther Deliv.

[153] Cao Z, Gao H, Xu Y (2025). Self-Assembled Nanoplatform with pH/NIR Light-Responsive Drug Delivery for Combined Therapy of Glioma in vitro. Int J Nanomedicine.

[154] An HW, Hou D, Zheng R, Wang MD, Zeng XZ, Xiao WY (2020). A Near-Infrared Peptide Probe with Tumor-Specific Excretion-Retarded Effect for Image-Guided Surgery of Renal Cell Carcinoma. ACS Nano.

[155] Hu Q, Katti PS, Gu Z (2014). Enzyme-responsive nanomaterials for controlled drug delivery. Nanoscale.

[156] Hao X, Zhang H, Yang Y, Lin Z, Bao H, Huang X (2026). Enzyme-responsive biomimetic ferritin nanoparticles for selective cancer therapy. Biomaterials.

[157] Sahu K, Firdous A, Raza MA, Saoji SD, Patravale VB, Ajazuddin (2025). Enzyme-responsive natural nanocarriers for RNA delivery in the tumor microenvironment: A comprehensive review. Journal of Drug Delivery Science and Technology.

[158] Fathi M, Safary A, Barar J (2020). Therapeutic impacts of enzyme-responsive smart nanobiosystems. Bioimpacts.

[159] Roszkowski S, Durczyńska Z, Szablewska S (2024). Targeted nanodelivery systems for personalized cancer therapy. Rep Pract Oncol Radiother.

[160] Zhang W, Mehta A, Tong Z, Esser L, Voelcker NH (2021). Development of Polymeric Nanoparticles for Blood-Brain Barrier Transfer-Strategies and Challenges. Adv Sci (Weinh).

[161] Wang C, Zhang W, He Y, Gao Z, Liu L, Yu S (2021). Ferritin-based targeted delivery of arsenic to diverse leukaemia types confers strong anti-leukaemia therapeutic effects. Nature Nanotechnology.

[162] Kim JH, Bae C, Kim MJ, Song IH, Ryu JH, Choi JH (2020). A novel nucleolin-binding peptide for Cancer Theranostics. Theranostics.

[163] Mirzayans R, Murray D (2022). What Are the Reasons for Continuing Failures in Cancer Therapy? Are Misleading/Inappropriate Preclinical Assays to Be Blamed? Might Some Modern Therapies Cause More Harm than Benefit?. International journal of molecular sciences.

[164] da Cunha NB, Cobacho NB, Viana JFC, Lima LA, Sampaio KBO, Dohms SSM (2017). The next generation of antimicrobial peptides (AMPs) as molecular therapeutic tools for the treatment of diseases with social and economic impacts. Drug Discov Today.

[165] Lei ZN, Tian Q, Teng QX, Wurpel JND, Zeng L, Pan Y (2023). Understanding and targeting resistance mechanisms in cancer. MedComm (2020).

[166] Wu J, Ji S, Sahibzada KI, Lou M, An F, Li W (2025). Artificial intelligence-driven anticancer peptide discovery. IMetaOmics.

